# Whole genome analysis of water buffalo and global cattle breeds highlights convergent signatures of domestication

**DOI:** 10.1038/s41467-020-18550-1

**Published:** 2020-09-21

**Authors:** Prasun Dutta, Andrea Talenti, Rachel Young, Siddharth Jayaraman, Rebecca Callaby, Santosh Kumar Jadhav, Velu Dhanikachalam, Mayakannan Manikandan, Bhim B. Biswa, Wai Y. Low, John L. Williams, Elizabeth Cook, Phil Toye, Eileen Wall, Appolinaire Djikeng, Karen Marshall, Alan L. Archibald, Suresh Gokhale, Satish Kumar, David A. Hume, James G. D. Prendergast

**Affiliations:** 1 The Roslin Institute, University of Edinburgh, Easter Bush, Midlothian, EH25 9RG UK; 2 Centre for Genomic and Experimental Medicine, MRC Institute of Genetics & Molecular Medicine, University of Edinburgh, Crewe Road, Edinburgh, EH4 2XU UK; 3 Centre for Tropical Livestock Genetics and Health, Easter Bush, Midlothian, EH25 9RG UK; 4 BAIF Development Research Foundation, Central Research Station, Uruli Kanchan, Pune, Maharashtra 412202 India; 5 Centre for Cellular and Molecular Biology, Habsiguda, Uppal Road, Hyderabad, Telangana 500007 India; 6 Davies Research Centre, School of Animal and Veterinary Sciences, University of Adelaide, Roseworthy, SA 5371 Australia; 7 Dipartimento di Scienze Animali, della Nutrizione e degli Alimenti, Catholic University of the Sacred Heart, Piacenza, Italy; 8 International Livestock Research Institute (ILRI), Nairobi, 30709-00100 Kenya; 9 Centre for Tropical Livestock Genetics and Health, ILRI Kenya, Nairobi, 30709-00100 Kenya; 10 Scotland’s Rural College (SRUC), Easter Bush, Midlothian, Edinburgh, EH25 9RG UK; 11 Department of Biotechnology, School of Life Science, Central University of Haryana, Mahendergargh, Haryana, 123031 India; 12 Mater Research Institute-University of Queensland, Translational Research Institute, Woolloongabba, QLD 4102 Australia

**Keywords:** Evolutionary genetics, Population genetics, Animal breeding, Genomics

## Abstract

More people globally depend on the water buffalo than any other domesticated species, and as the most closely related domesticated species to cattle they can provide important insights into the shared evolutionary basis of domestication. Here, we sequence the genomes of 79 water buffalo across seven breeds and compare patterns of between breed selective sweeps with those seen for 294 cattle genomes representing 13 global breeds. The genomic regions under selection between cattle breeds significantly overlap regions linked to stature in human genetic studies, with a disproportionate number of these loci also shown to be under selection between water buffalo breeds. Investigation of potential functional variants in the water buffalo genome identifies a rare example of convergent domestication down to the same mutation having independently occurred and been selected for across domesticated species. Cross-species comparisons of recent selective sweeps can consequently help identify and refine important loci linked to domestication.

## Abstract (web summary)

The comparative genomics of domesticated lineages can yield insights into the signatures of artificial selection. This study sequences 79 water buffalo genomes from 7 breeds and reveals examples of convergent domestication at the genetic level between water buffalo and cattle.

## Introduction

The domestic riverine water buffalo (*Bubalus bubalis*) is an important source of milk, meat and draft power in the Indian subcontinent^1^. Of the world population of ~200 million water buffalo, the majority are found in South Asia (FAOSTAT 2017 data^2^), where they provide more milk than cattle. The majority of these animals are owned by smallholder farmers in Asia, meaning more people depend on the water buffalo than any other domesticated species^3^. Two distinct groups of water buffalo exist, the river buffalo, which is found primarily in India and the swamp buffalo of East Asia^4^. Within the Indian subcontinent, phenotypic selection over many centuries has produced a number of well-defined regional breeds of river buffalo, displaying a diverse range of morphological and production phenotypes. Analysis of the maternal lineages of eight distinct breeds based upon mitochondrial D-loop region sequences^5^ indicated that breed differentiation and population expansion was relatively ancient, following domestication around 6300 years ago. Through migration and importation, water buffalo spread world-wide over the past 200 years, and have become an important commercial source of milk production in the Middle East and Mediterranean countries^6^. Analysis of 31 water buffalo populations across the world using a 90 K SNP Chip, which was designed on the basis of river buffalo sequence data^7^ aligned to the cattle genome, supported the view that the westward spread of river buffalo from India occurred in multiple independent waves^8^. The rapid decrease in the cost of genomic DNA sequencing has facilitated a revolution in the analysis of genotype–phenotype relationships in livestock and the identification of selective sweeps associated with performance traits and adaptation. For example, a recent analysis of tropically adapted African cattle breeds compared to commercial taurine breeds revealed signatures of selection for several aspects of tropical adaptation^9^, an important trait shared with water buffalo. The recently released chromosome-level assembly of the genome of a female Mediterranean water buffalo^10^ now provides the framework to address the fine detail of the genetic diversity of Indian water buffalo breeds and to identify candidate functional variation.

River buffalo have been selected for many similar traits as other domesticated species, for example size, coat colour and production phenotypes^11–13^. Consequently, understanding how selection for these traits has affected a particular species’ genome will likely not only provide insights into its phenotypic adaptation, but also that of other domesticated species. The extent to which domestication has driven convergent evolution across different species remains a relatively open question. Comparison of domesticated sheep and goats to their wild ancestors identified overlapping genomic locations of selective sweeps^14^, though the precise patterns of selection often varied. By extension, more recent selective sweeps between domesticated breeds of related species, selected for similar traits and resistance to shared pathogens, could also overlap, even though the underlying functional variants may be different. Characterizing functional variants associated with desired traits in one species may provide potential candidates for introduction into other species.

In this study, we sequence the genomes of 79 Indian water buffalo from 7 breeds from different locations in India and Europe and compare the selective sweeps identified to those found in specific breeds of other domesticated species, notably 294 animals of the related bovids, domestic cattle (*Bos taurus taurus, Bos taurus indicus*). The results highlight extensive genetic diversity within Indian river buffalo populations that provide the basis for future genomic selection^15^ for improvements in traits such as fertility, productivity and disease-resistance. We illustrate that many of the selective sweep intervals overlap those in other species, including individual candidate functional variants, providing evidence of convergent domestication across species.

## Results

### Water buffalo genome sequencing

To characterise the genetic diversity of Indian water buffalo we sequenced the genomes of 73 animals from six distinct Indian breeds: Banni, Bhadawari, Jaffarabadi, Murrah, Pandharpuri and Surti. These breeds cover a range of geographical regions across India and display phenotypic diversity in terms of their physical features, milk production and environmental adaptation (see Supplementary Fig. 1). For each of these breeds half of the animals were sequenced at an average coverage of 37× with the remainder sequenced at a mean of 8×. To enable comparisons with a distinct outgroup, six Mediterranean buffalo were also sequenced at a mean depth of 36×, leading to a final cohort of 79 animals (Supplementary Table 1). All sampling was done in accordance with the regulations of the relevant local research institutes (BAIF and ILRI) and ethics approval was obtained from The Roslin Institute’s and the University of Edinburgh’s Protocols and Ethics Committees. All animal work was carried out in accordance with the regulations of the Animals (Scientific Procedures) Act 1986.

The resulting DNA sequences were aligned to the high quality, chromosome-level reference assembly of the water buffalo (UOA_WB_1 assembly)^10^ and 37,682,631 single nucleotide variants (SNVs) and 5,897,230 short insertions/deletions (InDels) were detected. Following removal of low-quality variants (see ‘Methods’) we identified a final set of 26,247,559 biallelic SNVs of which 25,513,085 were autosomal.

### Water buffalo population structure

**Fig. 1:**
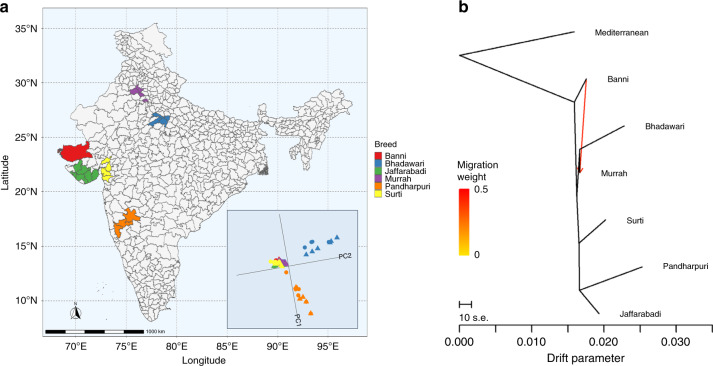
The relationship between genetics and geography among the Indian samples. **a** The main breeding tracts for each breed are indicated alongside an inset showing a principal component analysis of the genotype data. The genetic relationships largely mirror the geographic locations of the populations with the exception of the Murrah (purple) breed. **b** TreeMix analysis of the water buffalo breeds with the potential migration event indicated by an arrow (dark red indicates an event with stronger support).

Principal component analysis (PCA) of autosomal genotypes showed that the genetic relationship between the Indian breeds largely mirrors their geographic distribution (Fig. 1a). As expected, due to their comparative isolation, the Mediterranean animals are genetically distinct to the Indian animals (Supplementary Fig. 2). The Banni, Jaffarabadi and Surti breeds, which all originate from nearby areas of Western India, cluster together in the PCA. The relative geographic separation of the Pandharpuri and Bhadawari breeds is mirrored in their genomes, with these breeds being comparatively genetically distinct to the other breeds, as well as from each other. The exception to this relationship between geographic location and genetics is the Murrah breed, which although from Northern India was observed to be genetically similar to the West Indian breeds.

The extent of potential historical migration events between the populations is illustrated in Fig. 1b. Modelling the relationship between the populations using TreeMix^16^, the resulting tree could explain 99.26% of the variance between breeds. Apart from the separation from the Mediterranean animals, no deep splits were observed. There was strong support (*P* value < 1 × 10^−10^) for a mixture event between the Banni and Murrah populations, potentially providing an explanation for the observed closer genomic relationship between these breeds than expected given their geographic distribution.

### Mapping signatures of selection in the water buffalo genome

**Fig. 2:**
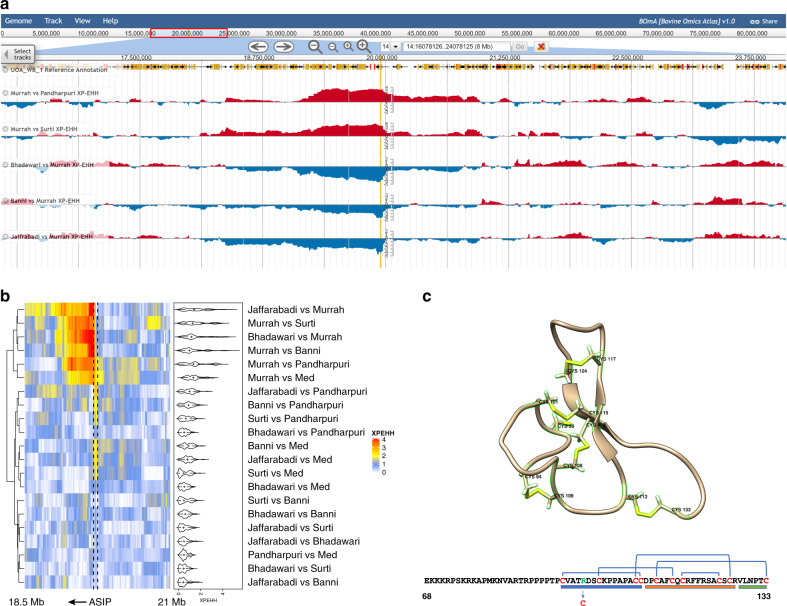
Patterns of selection at the *ASIP* locus. **a** XP-EHH scores across the *ASIP* locus as viewed in the BOmA browser (https://tinyurl.com/boma-asip). The location of the non-synonymous *ASIP* variant is indicated by a vertical orange line spanning all tracks. The elevated XP-EHH scores at this locus in comparisons involving the Murrah breed is shown. Low absolute XP-EHH scores are shown in blue with high scores in red. **b** Patterns of XP-EHH across all breed comparisons. Each column in the heatmap represents a different variant in the region, ordered according to their location in the genome. The intensity of colour indicates the variant’s XP-EHH score and the location of the *ASIP* gene is indicated by the dashed vertical lines. The distribution of absolute XP-EHH scores in each breed comparison across the region are indicated by the violin plots. **c** The location of the arginine to cysteine change in the cysteine rich agouti domain of ASIP. The location of the five disulphide bonds are indicated in both the predicted 3D structure and sequence below. The blue, orange and green boxes below the sequence indicate the locations of the N-terminal loop, active loop and C-terminal loop, respectively. The source data for panels (**a**) and (**b**) can be downloaded from the BOmA website.

To understand how genetic adaptation has contributed to the phenotypic diversity among buffalo breeds we characterized differences between populations using the cross-population extended haplotype homozygosity statistic^17^ (XP-EHH) and the cross-population composite likelihood ratio test^18^ (XP-CLR). These complementary statistics are widely used to determine the location of putative selective sweeps between populations of a species. XP-EHH is based on the fact that an allele under strong selection is expected to be swept to a high frequency faster than recombination can break down the haplotype upon which it resides. Consequently, elevated haplotype homozygosity in one population versus another is suggestive of differential selective pressures between the breeds^17^ providing a powerful approach to compare selection across the breeds of a domesticated species. The XP-CLR statistic is based on identifying where the change in allele frequency of variants in a region between populations has occurred more quickly than expected under random drift. XP-EHH and XP-CLR scores obtained from the 21 breed comparisons can be viewed along the water buffalo genome alongside the variant calls for each breed at a custom genome browser we have implemented at www.bomabrowser.com (Fig. 2a). In total there were 118 and 924 loci that showed elevated XP-EHH and XP-CLR scores, respectively in one or more breed comparison (see ‘Methods’, Supplementary Data 1–2). There was a substantial, significant overlap (permutation *P* = 1.3 × 10^−16^) in the loci detected by each method, with 62% of the XP-EHH loci at least partially overlapped by one of the XP-CLR peaks. This is approximately twice as many as expected by chance given the size and number of observed peaks. As shown in Supplementary Data 3, this enriched overlap was not specific to the chosen peak-calling parameters. Several of these loci contain genes that are putatively under selection or linked to key traits in other species. These include the fat-mass and obesity (*FTO*) gene which is strongly linked to body-weight traits in humans and cattle^19^, two genes (*KITLG* and *ASIP*) commonly linked to pigmentation/coat colour (see below), *GHR*, which has been linked to various phenotypes including milk composition in cattle^20^ and birth weight in humans^21^, and *HMGA2*, which has been associated with stature in both cattle^22^ and humans^23^.

### Convergent domestication at the ASIP pigmentation gene

Evidence for a putative strong selective sweep was observed at the *ASIP* (agouti signalling protein) locus on chromosome 14 (Supplementary Data 1). This gene has been linked to skin pigmentation in both humans^24^ and mice^25^ and was primarily associated with unusually high levels of haplotype homozygosity in the Murrah breed (Fig. 2b). Only two coding variants were detected in the *ASIP* gene, a synonymous variant at chr14:19,947,421 and a non-synonymous variant at chr14:19,947,429. The alternative allele at the synonymous variant was not observed in the Murrah, Surti or Mediterranean breeds and the highest frequency of 18% was observed in the Pandharpuri population. The frequency of this variant was therefore not correlated with the patterns of haplotype homozygosity at this region. In contrast, the alternative allele frequency of the non-synonymous variant was substantially higher (62.5%) among the Murrah animals compared to other breeds (from 4.5% in Surti to 20.8% in Banni). This variant leads to an arginine to cysteine amino acid change in the C-terminal agouti domain of the protein that is linked to melanocortin receptor binding activity in vitro^26^. This domain contains ten cysteine residues that form a network of five disulphide bonds shaping the active domain^27^ (Fig. 2c). The creation of an extra cysteine residue within this region consequently has the potential to disrupt the looping of this domain and its active site. Despite the arginine at this position being conserved across mammalian reference genomes (Supplementary Fig. 3), this arginine to cysteine change is exactly the same and at the orthologous position as a polymorphism in the dog ASIP protein that has been associated with the recessive inheritance of a uniform black coat among German Shepherds^28^. This suggests the same mutation has occurred independently in these two divergent domesticated species.

Variation at the *KITLG* gene, which controls melanocyte differentiation and migration, has also been associated with coat/skin colour in cattle^29^ and other species^11,30^ and both elevated XP-EHH and XP-CLR scores were observed upstream of *KITLG* in various comparisons of water buffalo breeds (Supplementary Fig. 4A). There are at least two distinct patterns of haplotype homozygosity in the vicinity of *KITLG* (Supplementary Fig. 4B). Comparison between the Banni and Jaffarabadi breeds indicated elevated XP-EHH in a region immediately upstream of the *KITLG* transcriptional start site, whereas the Pandharpuri breed shows a more extended region of elevated XP-EHH upstream of the gene. Variation in the receptor for KITLG (KIT) is associated with the proportion of black in Holstein cattle^31^ and a missense mutation in *KITLG* is associated with the roan cattle phenotype^29^. However, only one non-synonymous variant was identified in the water buffalo *KITLG* gene -chr4:101,938,991 found in only two Jaffarabadi animals. Accordingly, the putative selective sweep at this gene in water buffalo is potentially associated with transcriptional regulation.

### Selective sweeps linked to production phenotypes

**Fig. 3:**
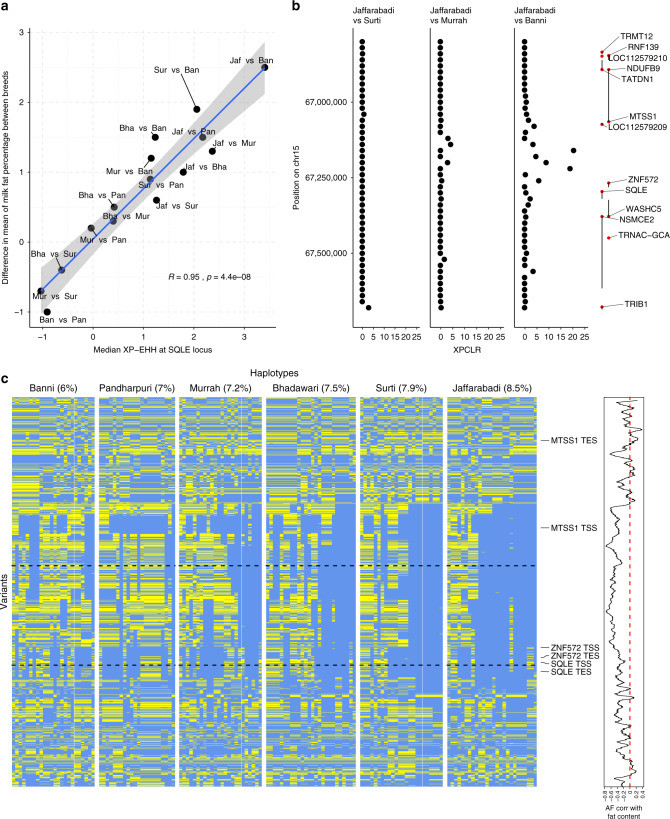
Patterns of selection at the *SQLE* locus. **a** The correlation between the median XP-EHH scores at the *SQLE* locus and the corresponding differences in milk fat percentages between each pair of breeds. The correlation and associated *P* value are indicated. The grey shaded area corresponds to the 95% confidence interval. **b** The XP-CLR scores at this locus in selected breed comparisons. The locations of genes in this region are indicated on the right with their TSS marked by red dots. **c** Haplotype homozygosity in each breed at this locus. Each column in the heatmaps represent a different haplotype with alleles colour coded according to their frequency in the Jaffarabadi breed (major allele in Jaffarabadi: blue, minor allele: yellow). The milk fat percentage is shown next to each breed name. The Jaffarabadi breed has a distinct elevated pattern of extended haplotype homozygosity at this locus. The horizontal dotted lines indicate the region spanned by the XP-CLR peak. The line graph to the right of the plot indicates the correlation between each variant’s frequency and milk fat percentage. Elevated correlations are observed specifically at the core region of this locus upstream of *SQLE*. The source data for these plots is available from the BOmA browser and Supplementary Table 2.

To provide an indication of which phenotypes may potentially underlie other selective sweeps, we correlated the median XP-EHH score across each locus for each breed comparison to the difference in mean breed value for nine different traits (wither height and birth weight in both males and females, lactation length, milk yield per lactation, milk fat content, age at first parturition and parturition interval). The strength of putative selection at one locus remained significantly associated with a trait after correcting for multiple testing (uncorrected Permutation *P* < 2 × 10^−4^, Bonferroni-corrected *P* < 0.024. Supplementary Data 4). This locus mapped to a 391Kb region on chromosome 15 and was significantly associated with milk fat percentage (Fig. 3a). It was also detected in the XP-CLR analysis (Fig. 3b) and contains five genes: *MTSS1*, *LOC112579209*, *ZNF572*, *SQLE* and *WASHC5*. *SQLE* is a strong candidate in this region due to its role in cholesterol biosynthesis, with the peak of selection falling in an intergenic region upstream of this gene (Fig. 3c).

A number of further loci were nominally associated with a trait and may point towards potential candidate traits for further investigation. For example, the second strongest association was at a region on chromosome 11 upstream of the *THBS1* gene where the XP-EHH scores were correlated with the differences in mean parturition intervals between breeds (uncorrected *P* = 0.0012). *THBS1* has previously been linked to ovarian cell development and function in cattle via the regulation of angiogenesis^32^. The *GHR* locus was most strongly associated with male and female birth weights (uncorrected *P* values of 0.016 and 0.019, respectively) and a common polymorphism affecting alternative splicing of the human *GHR* gene has previously been associated with differences in foetal growth and birth weight^21^.

### Cattle selective sweeps are preferentially linked to stature

**Fig. 4:**
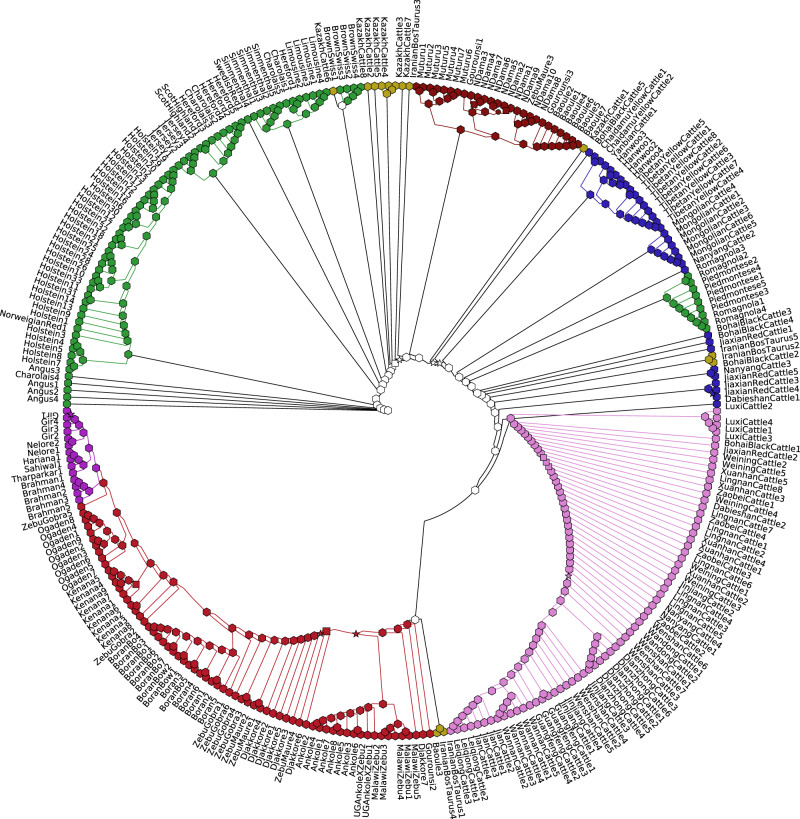
Identity by state phylogenetic tree of the set of 294 cattle whole genomes used in this analysis. The tree represents the IBS-based distance between samples when restricting to the 8,339,626 biallelic variants with a minor allele frequency >5%. Nodes are colour coded by the geographic origin of the animals (red: African indicine, purple: subcontinent indicine, pink: East-Asian indicine, blue: East-Asian taurine, green: European taurine, brown: African taurine, yellow: Central Asian taurine) and the marker shape represents the confidence in that node (Hexagon: confidence of 90–100%, star: 75–90%, square: 50–75%, circle: <50).

To investigate the extent to which the genomic regions under selection between water buffalo breeds may overlap those in another closely related domesticated species, we gathered whole-genome sequencing data for 427 cattle. This included a novel dataset of 82 African samples to increase the coverage of this continent that is comparatively under-represented in previous studies. These samples span a diverse range of breeds and geographic locations (Fig. 4 and Supplementary Fig. 5). Of note is the very deep split between the East Asian and other indicine cattle. Following quality control and the removal of related samples (see ‘Methods’) 294 distinct samples and 11,215,339 million variants were retained.

**Fig. 5:**
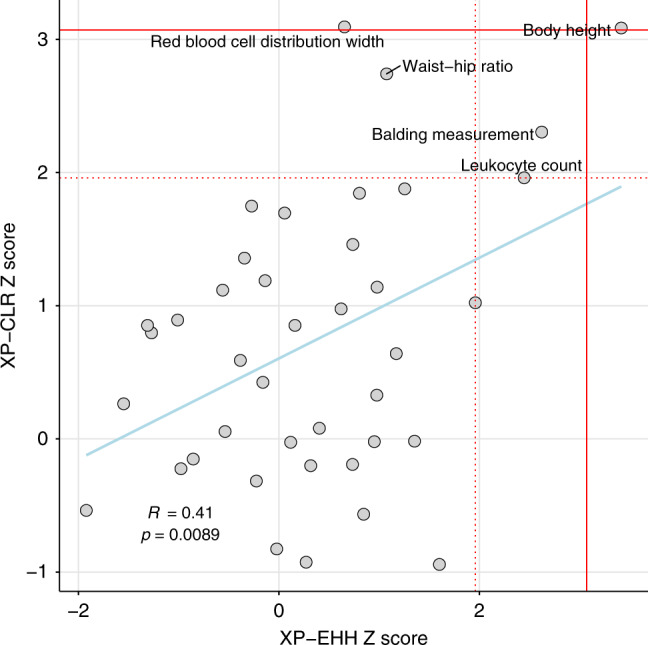
Cattle selective sweep enrichment analyses. Testing for an enrichment of the number of cattle selective sweep loci that overlap loci previously linked to phenotypes in human GWAS studies. The Z score represents the relative enrichment (or depletion if negative) of the number of the putative selective sweep intervals overlapping loci linked to the corresponding trait relative to randomly selected genomic regions of the same length. Each dot represents a different trait and the red dashed and solid lines represent the Z score thresholds that correspond to uncorrected and Bonferroni-corrected two-tailed *P* values of 0.05, respectively. The source data and results for all traits are shown in Supplementary Table 3.

To investigate between-breed signatures of selection we calculated XP-EHH and XP-CLR scores between each pair of cattle breeds with at least six unrelated samples (Ankole, Baoule, Boran, Brahman, Holstein, Iranian, Kazakh, Kenana, Lingan, Muturu, NDama, Ogaden and Tibetan Yellow) as for the water buffalo data. At the same cut-offs as used in the water buffalo analysis, 184 sites of elevated XP-EHH and 1867 sites of elevated XP-CLR (Supplementary Data 5–6) were observed between at least one breed pair (see ‘Methods’), covering a total of 149 and 418 Mb, respectively. As with the water buffalo data there was a significant overlap among the regions identified by each statistic with 139 (76%) of the XP-EHH regions overlapped by an XP-CLR peak (permutation *P* = 8.8 × 10^−14^, 93 expected to be overlapped by chance given the number and size of windows (standard deviation of mean = 6.13)). These regions spanned genes including *KIT*, which has been associated with coat colour, and myostatin (*MSTN*), linked to muscle development, but to test whether certain phenotypes are preferentially associated with these sites we first determined whether they preferentially overlapped genomic regions associated with phenotypes in human genome-wide association studies (GWAS). The cattle selective sweep peaks of both metrics were found to overlap regions that have been linked to body height in humans more often than expected by chance (Fig. 5, Supplementary Table 3). We observed that these peaks also overlapped genetic variants previously linked to stature specifically in cattle^22^ more often than expected (permutation-derived Z score *P* value for XP-EHH peaks: 0.05; for XP-CLR peaks: 0.002). The GWAS loci for four further traits showed at least a nominally significant overlap with putative selective sweep peaks (Fig. 5, Supplementary Table 3), with balding measurements and leucocyte count nominally significant in both the XP-EHH and XP-CLR analyses.

### Colocalisation of selective sweeps across bovids

**Fig. 6:**
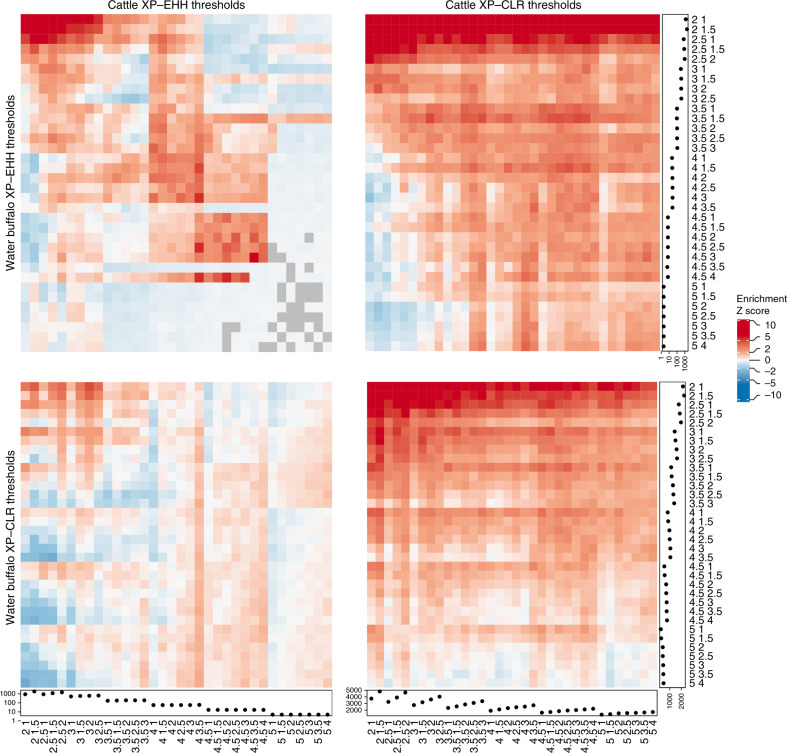
Overlap of peaks between species and metrics. Each heatmap shows a combination of metrics and the permutation-derived Z score of the enrichment (red) or depletion (blue) of overlapping peaks between species. Each row and column is labelled with the peak-calling parameters, the first number in the labels corresponds to the threshold that needed to be exceeded to call a peak and the second number the threshold that the metric needed to fall below to define the ends of the peak. The dot plots indicate the number of peaks called at each threshold. A Z score of 2 (or −2) corresponds to a nominal, uncorrected two-sided *P* value of ~0.05. The source data and associated *P* values are provided in Supplementary Data 7.

Using these cattle and water buffalo data we examined whether the locations of between-breed selective sweeps are shared across these domesticated bovid species. To do this, we lifted the water buffalo regions over to their orthologous locations in the cattle genome. We then examined whether the regions overlapped between the four combinations of species and metrics (XP-EHH or XP-CLR) more often than expected by chance. This was determined by repeatedly randomly sampling the same number and size of genomic regions from the cattle genome. To ensure this overlap was not just dependent on the chosen peak-calling parameters, we ran this overlap analysis across a wide range of peak-calling thresholds. As shown in Fig. 6, there is a significant enrichment of overlapping loci between species across most reasonable thresholds and metric comparisons, though with the lowest overlap observed between the XP-EHH cattle regions and XP-CLR water buffalo peaks. We can conclude that the location of between-breed selective sweeps within the two different species preferentially effect overlapping genomic regions.

To enable researchers to view and compare scores across these species, all cattle XP-EHH and XP-CLR scores lifted over to the water buffalo genome have also been uploaded to our browser at www.bomabrowser.com/waterbuffalo.

Using the random sampling data we were able to estimate how many regions are expected to overlap by chance in each comparison, and consequently how many of the overlapping regions are potentially false positives. Using these data, we identified thresholds for these comparisons that minimised the number of expected false positives (see ‘Methods’). At these thresholds, 4 out of 25 genomic loci identified in the water buffalo XP-EHH analysis that were successfully lifted over to the cattle genome overlapped regions identified in the cattle XP-EHH analysis (permutation *P* = 3.9 × 10^−6^), with less than one overlap expected by chance between these regions (Supplementary Data 7). A total of 22 of these same 25 regions identified in the water buffalo XP-EHH analysis overlapped a cattle XP-CLR peak (permutation *P* = 2.5 × 10^−4^, 13 regions expected to overlap by chance), including all of the four overlapping the cattle XP-EHH peaks.

**Fig. 7:**
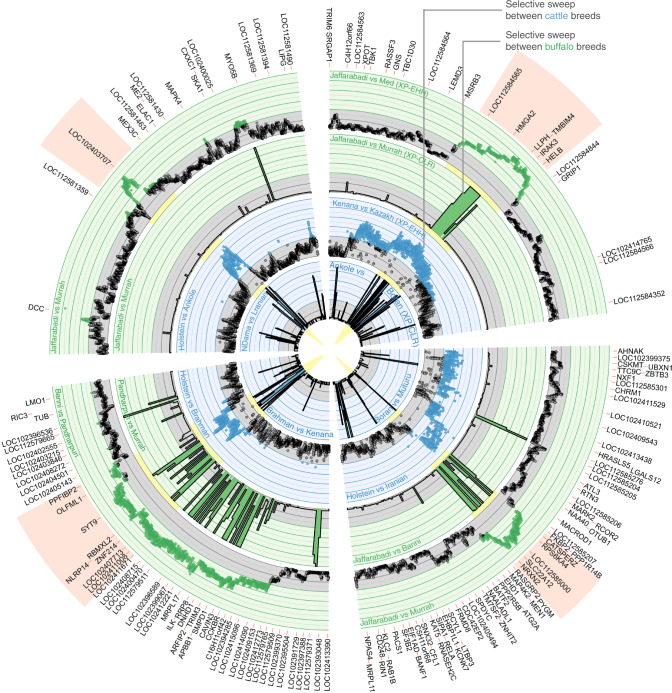
Colocalisation of between-breed selective sweeps in the water buffalo (green) and cow (blue) genomes at three genomic locations. The four discussed regions displaying overlapping putative selective sweep peaks between both water buffalo and cattle breeds are shown. An XP-EHH (outer) and XP-CLR (inner) track is shown for each species illustrating the overlap across metrics where applicable. XP-CLR scores were truncated at 6. For the two loci where only a subset of the four metrics produced overlapping peaks a random breed comparison is shown in the remaining tracks. The cattle scores were lifted over to their orthologous positions on the water buffalo genome to enable their direct comparison. The source data can be downloaded from the BOmA website.

Two of these four regions were detected in all four analyses, i.e. in both species and using both metrics. The first region was on cattle chromosome 5 (5:47374250–48270364) with the orthologous buffalo region on chromosome 4 (4:71990206–72881832) (Fig. 7). This locus contains two candidate genes underlying the selective sweeps; *HMGA2* that was one of the leading loci associated with stature in a recent meta-analysis of over 58,000 cattle^22^, and *IRAK3* that regulates toll-like receptor signalling and innate immune response^33^. As discussed above, selection for stature is likely one of the key drivers of differentiation between cattle breeds. However, the breeds linked to elevated XP-EHH scores at this locus are not consistently unusual in their stature (Supplementary Table 2). This suggests responses to pathogens, which will affect livestock across geographic regions, may be the stronger candidate for having driven selection at this locus. The second locus detected in all four analyses (i.e. in both species with both metrics) was on buffalo chromosome 16 (16:37,462,261–39,835,982), cattle chromosome 15 (15:45,078,520–47,503,196). This locus contains a large number of genes though only three with an orthologue in both species that was overlapped by the peaks called in all four analyses. These were *NLRP14*, *RBMXL2* and *SYT9*. A third region had overlapping peaks in three out of four comparisons, the exception being the cattle versus water buffalo XP-CLR analyses. This region, mapping to chromosome 5 (5:119,150,450–119,690,905) of the water buffalo genome (29:42,512,413–43,051,989 on the cow), contains 8 genes with orthologues in both water buffalo and cattle (*GPR137, KCNK4, CATSPERZ, ESRRA, TRMT112, PRDX5, CCDC88B and RPS6KA4*) and has been associated with body mass index in humans^34^. The final locus with overlapping peaks in two out of four comparisons (water buffalo XP-EHH peaks versus both cattle XP-EHH and XP-CLR peaks at water buffalo: 22:11,073,687–11,463,868; cow: 24:50,745,532–51,131,347) contained no annotated protein-coding genes but overlaps a lncRNA of unknown function and is directly upstream of *MEX3C* that has been linked to growth hormone regulation^35^.

## Discussion

Water buffalo and cattle are estimated to have last had a common ancestor over 5.8 million years ago^36^. However, since domestication humans have exerted strong artificial selection on both species for similar traits. In this study, we have shown that there is a core set of loci under apparent differential selection between breeds in both species. This core set of loci likely extends beyond just the bovinae, e.g. the *HMGA2* and *GHR* loci, that are both under apparent selection between cattle and water buffalo breeds, were also detected in a recent analysis of the genetic loci underlying differences in height between dog breeds^37^. Previous studies have suggested an overlap in the genomic regions targeted by selection over the comparatively long time-scales between wild and domesticated animals^14^. Here we show how signatures of selection between domesticated breeds within a species are also shared across species genome-wide. Consequently, ongoing selective pressures are likely still targeting overlapping loci across species. Identifying the causative variations underlying selective sweeps is difficult due to the large regions identified but overlapping analyses across species may help to narrow the intervals. Although the precise genetic variants at these loci that are under selection are expected to most often differ between species, we have identified that at least in some instances (e.g. *ASIP*) the exact same change appears to be under selection. This provides a rare example of apparent convergent domestication in mammals at the level of the individual base pair and amino acid change. Where the same loci, but different underlying variants, are under selection across species, there may be the potential to introduce the genetic change from one species to the other, using modern gene editing techniques, to accelerate selection for the trait.

With the advent of whole-genome sequencing, studying signatures of selection within a species genome-wide is now relatively routine. However, understanding which trait is driving the patterns of selection at a locus remains challenging. Especially as many genes and loci can be associated with a variety of potential traits, with the *GHR* locus, observed to be under selection in this study, linked to a range of fertility^38^, milk^20^ and meat^39^ production phenotypes in cattle. We show though that the strength of selection at these loci can be strongly correlated to the differences between breeds of certain traits, providing potential clues as to the driving forces behind the selective sweeps. For example, XP-EHH scores between buffalo breeds at the *SQLE* locus are strongly correlated to differences in the breeds’ milk fat percentages. A number of loci also overlap regions linked to various relevant traits in human GWAS studies providing further indications of the putative traits linked to a region. As well as the links to stature observed among cattle peaks, a number of peaks common to both bovid species are linked to other traits and disorders. These include peaks spanning *PTPN22, PRDX5* and *IRF4* linked to vitiligo^40^ and alopecia and *ALB* linked to obesity^41^.

We present an analysis of a whole-genome sequence dataset of water buffalo breeds, an accompanying browser to visualise selective sweeps across the water buffalo genome and a genome-wide comparison with selective sweeps across thirteen breeds of cattle. These data have the potential, to not only inform future improvement of water buffalo breeds but show that some selective sweeps are relevant across diverse domesticated species.

## Methods

### Water buffalo read alignment, variant calling and filtering

Paired-end sequencing of 81 animals was done across two sequencing centres: Edinburgh Genomics in the UK and SciGenom Labs in India. Edinburgh Genomics (UK) prepared the sequencing libraries using the Illumina TruSeq Nano DNA Library Prep Kit and sequencing was done using the Illumina HiSeq X platform (read length: 150 bp). SciGenom Labs (India) used the NEBNext Ultra DNA Library Prep Kit for library preparation and the Illumina HiSeq 2500 platform (read length: 250 bp) for sequencing. Eighty-one paired-end Illumina water buffalo DNA-seq samples were aligned to the water buffalo reference genome (UOA_WB_1 assembly)^10^ using BWA-MEM^42^ v0.7.17. For each sample, the BWA-MEM software generated sequence alignment map^43^ (SAM) output which was converted to binary alignment map^43^ (BAM) output using SAMtools^43^ ‘view’ v1.6 and then coordinate sorted using SAMtools ‘sort’ v1.6 with default parameters. Duplicate reads were marked using Picard (http://broadinstitute.github.io/picard) ‘MarkDuplicates’ v2.14.0. Read groups were added to each BAM file using Picard ‘AddOrReplaceReadGroups’ v2.14.0. GATK^44^ (The Genome Analysis Toolkit) HaplotypeCaller^45^ v4.0.4.0 was run per sample in GVCF (Genomic Variant Call Format file) mode using the parameter -ERC GVCF on the pre-processed BAM files. GATK’s GenomicsDBImport v4.0.4.0 was then used to aggregate all 81 GVCF files per scaffold. Then, GATK’s GenotypeGVCFs v4.0.4.0 with -new-qual parameter was used to perform joint genotyping and output the final multi-sample variant call format^46^ (VCF) file per chromosome/scaffold. Finally, Picard ‘GatherVcfs’ v2.14.0 was used to concatenate variants called per scaffold to get the final multi-sample VCF file containing SNVs and InDels. Only biallelic SNVs were retained using BCFtools^47^ ‘filter’ v1.6 with -v snps and -m2-M2 parameters.

Only those biallelic SNVs were kept which had a quality by depth (QD) ≥ 15, fisher strand bias (FS) ≤ 60, strand odds ratio (SOR) ≤ 2, root mean square mapping quality (MQ) ≥ 50, mapping quality rank sum test ≥ −2.5 and a read position rank sum test ≥ −2.5. These metrics were chosen to maximise the Ti/Tv (transitions vs. transversions) ratio whilst minimising the number of variants removed. After applying the filters on raw biallelic SNVs, the global Ti/Tv ratio increased from 2.01 to 2.29. The Ti/Tv ratio is often used as a quality indicator of variation data produced from next-generation sequencing (NGS) experiments. A higher Ti/Tv ratio is usually an indicator of good quality SNVs^48^ as sequencing errors and false positive variant calls have a Ti/Tv closer to one. The global or genome-wide estimate of Ti/Tv ratio for humans has been reported to be between 2.0 and 2.2^49^. The Ti/Tv ratio for WGS data of *Bos taurus* (cattle) has previously been found to be in a similar range as in this study^50^. The variant calling and filtration process resulted in a total of 26,247,559 high quality biallelic SNVs discovered across the water buffalo genome out of which 25,513,085 were autosomal. The final set of SNVs were annotated using SnpEff^51^ v4.3e.

### Water buffalo relatedness analysis

A relatedness analysis was undertaken using VCFtools^46^ v0.1.13 with the --relatedness2 parameter^52^ in order to infer sample relationships and as a quality control procedure to check if any samples were sequenced twice. Two duplicates were identified and among these, the samples with higher depth were retained, leaving 79 individuals. The duplicate samples are graded in yellow and green in Supplementary Table 1.

### Water buffalo population structure analysis

For the PCA (principal component analysis), PLINK^53^ v1.90b4 64-bit was used to generate the principal components (PCs) from the filtered autosomal biallelic SNVs which were then plotted using the ggplot2^54^ package in R^55^. Only those sites were kept which were genotyped in every sample, had a minor allele frequency (MAF) ≥ 5% and a phred scaled genotype quality of ≥20. The software TreeMix^16^ v1.13 was used to determine population splits and migration events amongst the populations involved in this study. The TreeMix algorithm was run for 7 migration events using the –m parameter. The maximum likelihood tree was constructed using blocks of 1000 SNVs to account for variants that are non-independent because they lie close to each other using the parameter -k 1000. The root was defined using the –root parameter considering the Mediterranean breed as an ‘outgroup’. The outputs were plotted using the R function ‘plot_tree’, which is included with the TreeMix software.

### Water buffalo selection signature analysis

Filtered biallelic SNVs from 79 samples were processed through PLINK^53^ v1.90b4 64-bit with a genotyping rate of at least 80%, a MAF ≥ 5%, and minimum phred scaled genotype quality of 20 to produce a VCF file consisting of 18,358,331 SNPs. This set of SNPs was then used with SNeP^56^ v1.1 with its default settings to estimate the effective population size (Ne) of the samples. The tool removed loci with missing values (used loci with 100% genotyping rate) and finally utilized 74,007 SNPs for Ne calculation. The Ne value for the most recent generation (13) was 358. The VCF consisting of 18,358,331 SNPs was further processed where missing genotypes were self-imputed using Beagle^57^ v5.0 with default parameters and all samples were utilised for imputation. Phasing was done using Beagle v5.0 with the Ne set to the estimated value of 358. In the absence of a good quality recombination map, the recombination rate was assumed to be 1 cM (centiMorgan) per Mbp for the water buffalo genome. Seven breed-wise VCF files (each VCF file contained individuals from only one breed) were produced from the phased and imputed VCF file using BCFtools^47^ v1.6, which were then converted to IMPUTE hap format (suitable for hapbin^58^) using VCFtools v0.1.13. Twenty-one pairwise XP-EHH tests were performed between all pairs of breeds (7 breeds in total, 21 unique combinations) using hapbin’s^58^ xpehh program v1.3.0. XP-EHH scores were smoothed by averaging across 1000 SNP windows and putative selective sweep regions were those with an absolute XP-EHH >3.5, the start and end coordinates defined where the XP-EHH scores fell back below 1.5. We repeated key analyses across a wide range of thresholds to ensure they were not specific to these cut-offs (Supplementary Data 3–4). The resulting Browser Extensible Data (BED) format files were then intersected with another bed file containing all the gene coordinates from the water buffalo gene annotation file in GTF (Gene transfer format) using BedTools^59^ ‘intersect’ v2.27.1 to get a list of candidate genes that may be under selection.

XP-CLR was calculated using the xpclr software v1.1.2 (https://github.com/hardingnj/xpclr) on the variant dataset prior to imputation, allowing only polymorphic sites with a minor allele frequency >5%. The window size was set to 50 Kb^9^ (--size 50,000) allowing up to 600 SNPs per windows (--maxsnps 600). To remove individual outliers XP-CLR values were averaged across the three flanking windows either side and putative selective sweep regions defined as those with values exceeding 4.5 with the start and end locations being where the values fell back below 2.5. Again, key analyses were repeated across a range of thresholds.

For both XP-EHH and XP-CLR analyses overlapping peaks were merged across breed comparisons. The BOmA browser for visualizing the XP-EHH and XP-CLR scores was implemented using JBrowse^60^.

### Association of selective sweeps with buffalo traits

Mean wither height and birth weight in both males and females, lactation length, milk yield per lactation, milk fat content, age at first parturition and parturition interval for each available water buffalo breed were downloaded from the FAO website (http://www.fao.org/dad-is/dataexport/en/). These metrics are provided in Supplementary Table 2. The Pearson’s correlation coefficient was then calculated between the difference of these values between all pairs of breeds and the corresponding median XP-EHH scores at each peak. To characterise the probability of getting as strong a correlation by chance 5000 permutations were run, where for each real peak random regions of the genome of the same size were randomly sampled and the correlations to their median XP-EHH scores calculated. The percentile at which the real correlation value fell within this distribution of permutation values was then calculated using the ecdf (empirical cumulative distribution function) function in R^55^ to determine the two-tailed *P* value. A Bonferroni correction was applied to these *P* values to account for the number of peaks tested. The final results are listed in Supplementary Data 4.

### Cattle sequence data analysis

Illumina sequencing data for 427 cattle genomes representing a wide diversity of global cattle breeds were aligned to the cattle reference ARS-UCD1.2^61^, extended with the Y chromosome from the Btau_5.0.1 assembly^62^, using BWA-MEM^42^ v0.7.17. This included 82 novel datasets from samples of African cattle breeds. The remaining cattle sequencing datasets can be found in online databases with the accession numbers listed in Supplementary Data 8. Aligned reads were then labelled with GATK^44^ PrintReads v4.0.11.0, multiple libraries for a single sample were merged with BamTools^63^ v2.4.2. Reads were sorted with SAMtools^43^ ‘sort’ v1.9, duplicates were marked (MarkDuplicates) with GATK v4.0.11.0 and base-quality score recalibration or BQSR (BaseRecalibrator and ApplyBQSR) was performed using the dataset provided by the 1000 bulls genome consortium at their website^64^ and the variants in the Illumina BovineHD BeadChip. Autosomal variants for each sample were called using GATK’s HaplotypeCaller^45^, and samples combined using the GenomicDBImport and GenotypeGVCFs functions in GATK v4.0.11.0. The whole pipeline can be recreated using BAGPIPE (https://bitbucket.org/renzo_tale/bagpipe/).

After defining the variants, GATK’s variant quality score recalibration (VQSR) approach was performed using multiple sources, including the BQSR file from the 1000 Bulls genome project^64^, 24 SNP chip datasets and variants from Ensembl^65^ v95 (ftp://ftp.ensembl.org/pub/release-95/variation/vcf/bos_taurus/). The parameters considered were mapping quality rank sum test (MQRankSum), Strand odd ratio (SOR), Fisher strand bias (FS), quality by depth (QD), the read position rank sum test (ReadPosRankSum) and the Inbreeding Coefficient (InbreedingCoeff). Further details on the VQSR stage are reported in Supplementary Note. InDels were filtered using the same hard filtering specified at the GATK web page (https://gatk.broadinstitute.org/hc/en-us/articles/360035531112--How-to-Filter-variants-either-with-VQSR-or-by-hard-filtering), i.e. QD > 2.0, FS ≤ 200.0, ReadPosRankSum ≥−20.0, SOR ≤ 10.0. After filtering variants in the 99% tranche, SNPs with QUAL > 100 and biallelic, a total of 64,447,506 variants were retained and annotated using both SnpEff^51^ v4.3t and the Ensembl Variant Effect Prediction^66^ (VEP) tool configured to define the deleterious variants by SIFT^67^. Read based phase produced by GATK were retained, where possible. Individuals with low call rate were removed from downstream analyses (individual call rate <97%, Supplementary Data 9). Variants were further screened to retain only high quality, low missingness genotypes. Multiple values of call rate (CR) and genotype quality (GQ) have been assessed in order to define the most balanced values to adopt for the downstream analyses, as shown in Supplementary Fig. 6 and Supplementary Table 4. After the evaluation of the dataset, we opted to keep variants with a CR ≥ 75% and GQ ≥ 25, which retained a total of 11,215,339 variants.

### Cattle population analysis

Highly related individuals (relatedness value from VCFtools^46^ --relatedness2 > 0.0625) and those with a high proportion (>25%) of missing genotypes were excluded. The identity by state (IBS) phylogenetic tree was generated using PLINK^53^ 1.90b4 64-bit, considering biallelic markers (both SNPs and InDels) with MAF ≥ 5%.

### Cattle selection signatures analysis

After identifying those breeds with at least six animals, and after filtering by relatedness, XP-EHH and XP-CLR were calculated between all combinations of the remaining breeds. Only SNPs were considered for the selection signatures discovery. XP-EHH was calculated using hapbin^58^ in its default settings as for the water buffalo data, with the exception that Ne was set to 1000 during the phasing with Beagle^57^ v5.0.

XP-CLR was calculated using the xpclr software v1.1.2 (https://github.com/hardingnj/xpclr) on the dataset prior to imputation, allowing only polymorphic sites (minor allele frequency >1%), 50Kb windows (--size 50,000) and allowing up to 600 SNPs per windows (--maxsnps 600). Peaks were called using the same approach and parameters as for the water buffalo data.

### GWAS enrichment analyses

The locations of single nucleotide polymorphisms linked to phenotypes in human GWAS^68^ were mapped onto the cattle genome using LiftOver^69^. For each phenotype with at least 300 variants the number of XP-EHH or XP-CLR peaks overlapping at least one GWAS variant was calculated. To determine if this was more or less than expected, the same number of regions of the same sizes were resampled from the cattle genome 1000 times. This therefore accounted for the number and distribution of GWAS variants along the genome, as these were kept unchanged across the permutations. The mean number of these randomized peaks overlapping the same GWAS variants across permutations was then used to calculate a Z (standard) score and associated *P* value.

To test for an association between the cattle peaks and stature GWAS variants identified specifically in cattle, we used the results from a 2018 study^22^. Enrichment of the peaks overlapping these GWAS variants was estimated in the same way as for the human GWAS variants above.

### Liftover from the water buffalo to the cow genome

To determine orthologous positions between the water buffalo and cattle genomes, we performed a liftover of the genomic positions using FLO^70^ (https://github.com/wurmlab/flo), which is a pipeline that uses the UCSC tools^71^, with the settings “-fastMap -tileSize=1 -minIdentity=90”. The minimum identity percentage was chosen to be similar to that used by Mintoo and colleagues for their LASTZ alignments^36^. Genomic positions were lifted from one genome to the other using the LiftOver^69^ software.

### Testing overlap between water buffalo and cattle

To test whether regions of elevated XP-EHH or XP-CLR in one species overlapped peaks in the other species more often than expected by random, we first lifted all water buffalo peaks over to the cow genome. After determining the real number of overlapping peaks, regions of the same number and sizes were sampled from the cow genome 100 times and the number of overlapping peaks recalculated. Using these permutation results we calculated a Z score for each comparison representing how many standard deviations the real number of overlaps was above the mean of the permutation results. These Z scores were then converted to *P* values and the results across peak-calling thresholds are shown in Supplementary Data 7. Using these results we identified new peak-calling thresholds that balanced the estimated false positive rates across analyses (maximum and minimum metric values of 4 and 1.5 for buffalo XP-EHH, 4.5 and 2 for buffalo XP-CLR, 4 and 3.5 for cattle XP-EHH and 5 and 2.5 for cattle XP-CLR).

### Reporting summary

Further information on research design is available in the Nature Research Reporting Summary linked to this article.

## Supplementary information

Supplementary Information

Peer Review File

Reporting Summary

Description of Additional Supplementary Files

Supplementary Data 1

Supplementary Data 2

Supplementary Data 3

Supplementary Data 4

Supplementary Data 5

Supplementary Data 6

Supplementary Data 7

Supplementary Data 8

Supplementary Data 9

Supplementary Software 1

## Data Availability

All XP-EHH and XP-CLR scores used in this analysis are viewable and downloadable at our BOmA browser (https://www.bomabrowser.com/waterbuffalo/). The raw sequencing data for the novel water buffalo and cattle samples have been deposited at the European Nucleotide Archive (ENA) with study IDs PRJEB39591, PRJEB39330 and PRJEB39924. The accessions for the previously published datasets can be found in Supplementary Data 8. Additional source data for Figs. 1b and 4 and Supplementary Fig. 2 are provided in the Source data and code file.

## Code availability

The code to repeat analyses is available at https://github.com/evotools/wBuffaloVsCow and alongside the input files at https://datashare.is.ed.ac.uk/handle/10283/3665.
