# Supplementary material for: Whole genome analysis of water buffalo and global cattle breeds highlights convergent signatures of domestication
Source: Nat Commun. 2020 Sep 21;11:4739. doi: 10.1038/s41467-020-18550-1 (PMC7505982; doi:10.1038/s41467-020-18550-1)
Supplement: Supplementary file 1 — Supplementary Information [file 41467_2020_18550_MOESM1_ESM.pdf]

Whole genome analysis of water buffalo and global cattle breeds highlights convergent signatures of domestication

Dutta, P., *et al.*

This PDF file includes:

1) Supplementary Figures 1-6
2) Supplementary Note
3) Supplementary Tables 1-4

Supplementary Figures

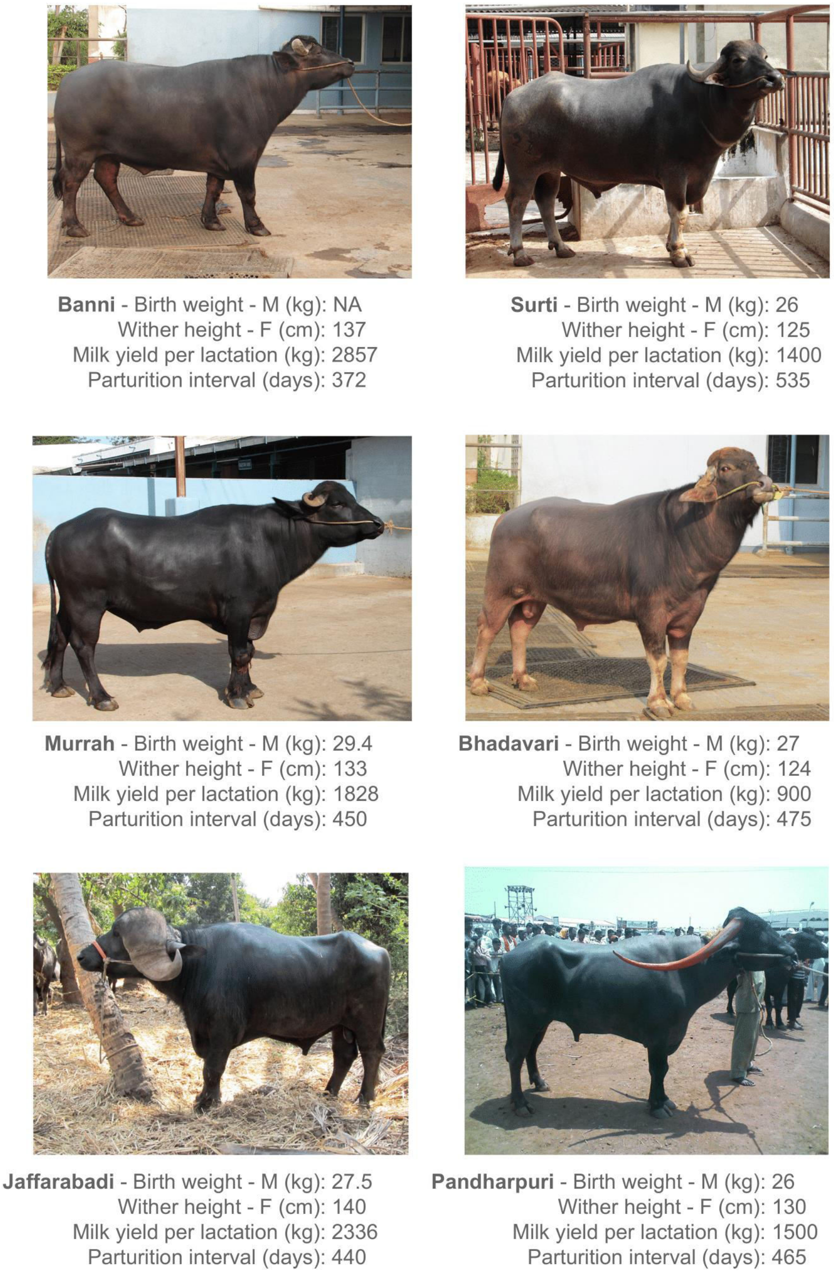

Supplementary Figure 1: Indian water buffalo breeds. The Indian water buffalo breeds sequenced in this study and their average phenotypic characteristics as recorded by the FAO.

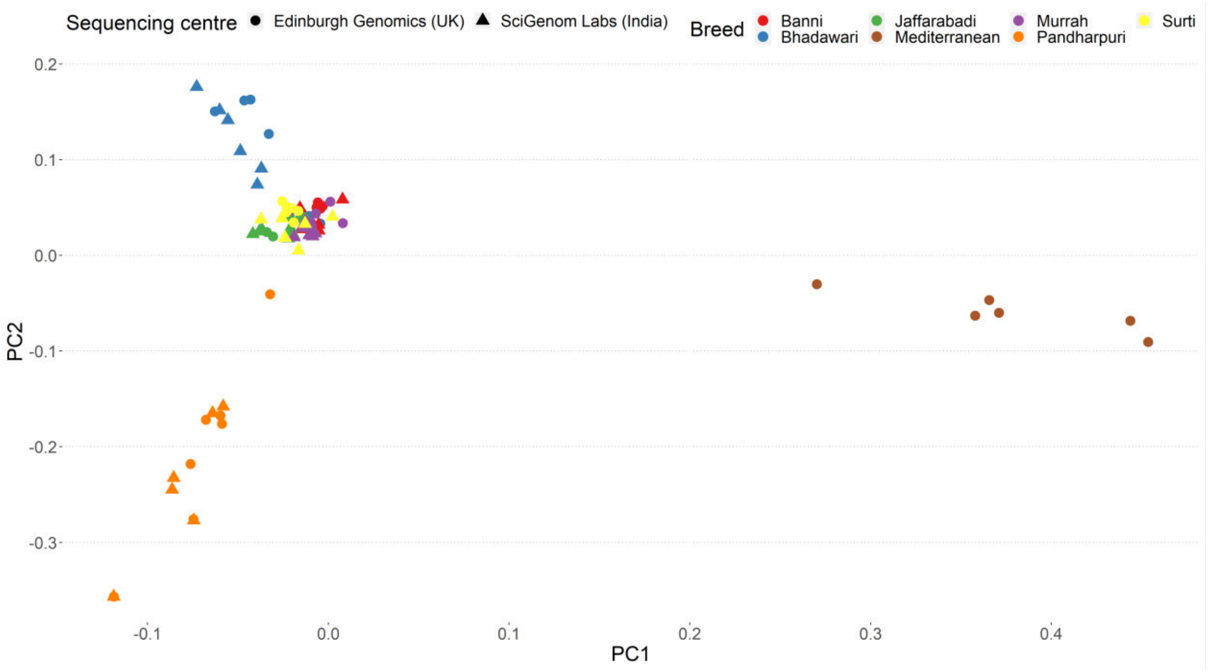

Supplementary Figure 2: Principal Component (PC) analysis, PC1 versus PC2. The plot explains the breed differences based on 64,954 variants from 81 animals from 7 breeds. The points in the plot signify each animal, with different colours denoting their respective breed and the shape of the points denoting their respective sequencing centre.

```
Canis_lupus_familiaris      MNIFRLLLATLLVSLCFLTAYSHLA-EEKPKDDRSLRSNSSVNLLDFPSVSIVALNKKSK     59
Homo_sapiens                MDVTRLLLATLLVFLCFFTANSHLPPEEKLRDDRSLRSNSSVNLLDVPSVSIVALNKKSK     60
Equus_caballus              MDVIHLFLATLLVSLCFLTAYSHLSPEEKPKDDRSLRNNSSMNLLDSPSVSIMALNKKSK     60
Sus_scrofa                  MDVTRLLLATLLVCLCFFTASSHLAPEEKSKDERSLRSNSSMNLLDFPSVSIVALNKKSK     60
Bos_mutus                   MDVSRLLLATLLVCLCFLTAYSHLAPEEKPRDERNLKNNSSMNLLDFPSVSIVALNKKSK     60
Capra_hircus                MDVSRLLLATLLVCLCFLSAYSHLAPEEKPRDERNLKNNSSMNLLDFPSVSIVALNKKSK     60
Bubalus_bubalis             MDVSRLLLATLLVCLCFLTAYSHLAPEEKPRDERNLKNNSSMNLLDFPSVSIVALNKKSK     60
Bos_taurus                  MDVSRLLLATLLVCLCFLTAYSHLAPEEKPRDERNLKNNSSMNLLDFPSVSIVALNKKSK     60
                            *:: :*:****** ***::* ***  *** :*:*.*:.***:**** *****:*******

Canis_lupus_familiaris      KISRKEAEK-KRSSKKKASMKNVARPRPPPPTPCVATRNSCKSPAPACCDPCASCQCRFF     118
Homo_sapiens                QIGRKAAEK-KRSSKKEASMKKVVRPRTPLSAPCVATRNSCKPPAPACCDPCASCQCRFF     119
Equus_caballus              KISRKEAEKKKRSSKKKASMTKVARPRLLQPAPCVATRDSCKPPAPACCDPCASCQCRFF     120
Sus_scrofa                  KISRKEAEK--RSSKKKASMKKVAQPRPPRPAPCVANRDSCKPPALACCDPCAFCQCRFF     118
Bos_mutus                   KISRNEAEKKKRPSKRKAPMKNVARTRPPPPTPCVATRDSCKPPASACCDPCAFCQCRFF     120
Capra_hircus                KISRNEAEKKKRASKRKAPMKNVARTRPPPPTPCVATRDSCKPPAPACCDPCAFCQCRFF     120
Bubalus_bubalis             KMSRNEAEKKKRPSKRKAPMKNVARTRPPPPTPCVATRDSCKPPAPACCDPCAFCQCRFF     120
Bos_taurus                  KISRNEAEKKKRPSKRKAPMKNVARTRPPPPTPCVATRDSCKPPAPACCDPCAFCQCRFF     120
                            ::.*: ***  * **::* *.:*.: *    :**** *:*** ** ******* ******

Canis_lupus_familiaris      RSACTCRVLSPRC--------------------   131
Homo_sapiens                RSACSCRVLSLNC--------------------   132
Equus_caballus              RSACSCRVLTRTC--------------------   133
Sus_scrofa                  RSACSCRVLNPTC--------------------   131
Bos_mutus                   RSACSCRALFFLPQGGGAGLPGPWGLSGVGELW   153
Capra_hircus                RSACSCRGLNPTC--------------------   133
Bubalus_bubalis             RSACSCRVLNPTC--------------------   133
Bos_taurus                  RSACSCRVLNPTC--------------------   133
                            ****:** *
```

Supplementary Figure 3: Multiple sequence alignment of ASIP protein. The figure shows conserved Arginine residue (red box) across various mammals.

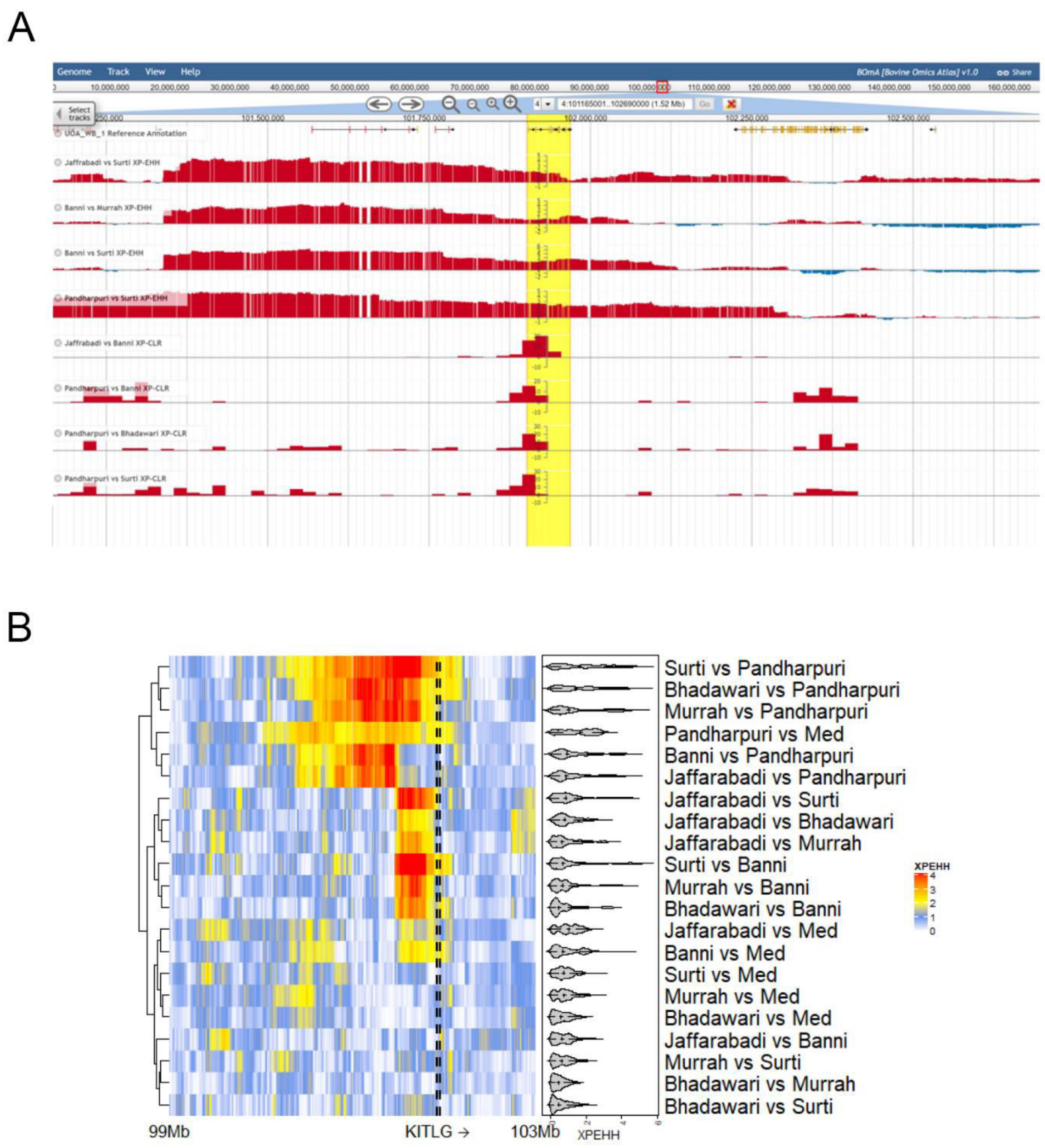

Supplementary Figure 4: Patterns of selection at the *KITLG* locus. A) XP-EHH and XP-CLR scores at the *KILTG* gene (highlighted in yellow) as shown in the BOmA browser. B) Each column in the heatmap represents a different variant in the region, ordered according to their location in the genome. The intensity of colour indicates the variant's XP-EHH score and the dashed vertical lines indicate the location of the *KITLG* gene.

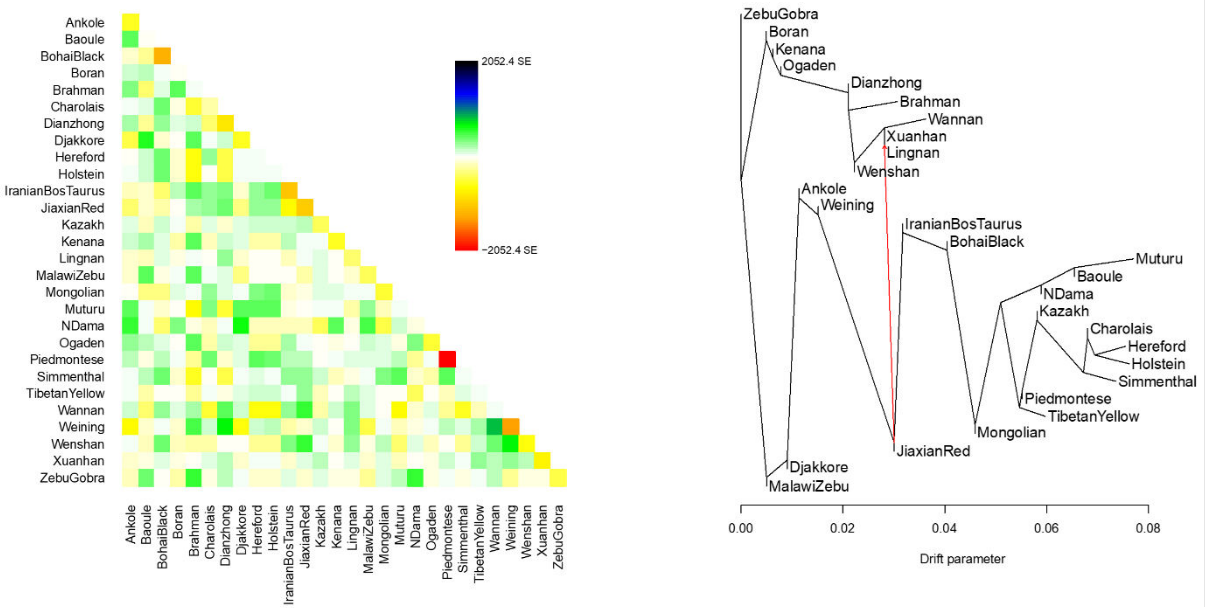

Supplementary Figure 5: Cattle TreeMix analysis. TreeMix maximum likelihood tree with one admixing event allowed (right) of the cattle breeds with the respective residual heatmap (left); the tree clearly shows the clear split between taurine (lower branch of the tree) and indicine breeds (upper branch of the tree).

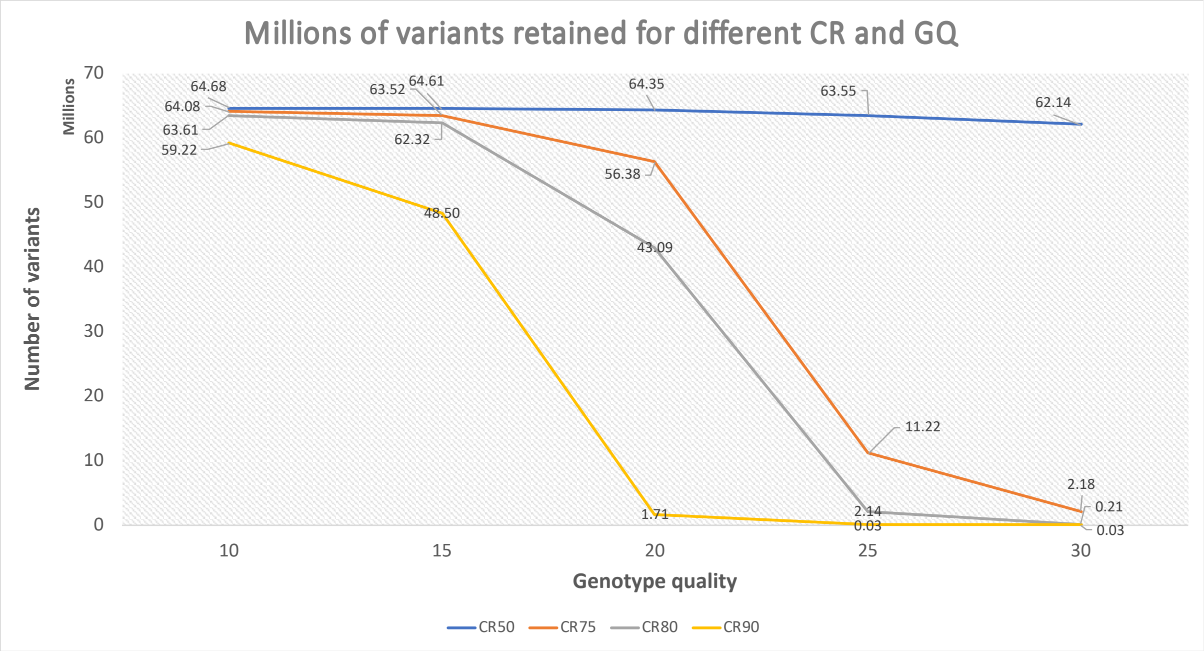

Supplementary Figure 6: Genotype quality (GQ) vs call rate (CR). Assessment of multiple genotype quality (GQ) and call rate (CR) filtering criteria for the cattle dataset. Data underlying this plot has been presented as Supplementary Table 4.

Supplementary Note - Variant quality score recalibration (VQSR)

Variant quality score recalibration has been performed using a total of 23 resources.

The BQSR variants can be downloaded from the 1000 bulls genome project website (http://www.1000bullgenomes.com/doco/ARS1.2PlusY_BQSR_v2.vcf.gz), the dbSNP file is now accessible from the Ensembl ftp website (ftp://ftp.ensembl.org/pub/release-95/variation/vcf/bos_taurus) and the 23 SNP chip information can be downloaded at the AnGenMap website (https://www.animalgenome.org/repository/cattle/UMC_bovine_coordinates/).

The command, inclusive of the resources used, to perform the VQSR is reported below:

```
gatk --java-options "-Xmx22g" VariantRecalibrator \
   -R ARS-UCD1.2_Btau5.0.1Y.fasta \
   -V input.vcf.gz \
   -resource
777HD,known=false,training=true,truth=true,prior=15.0:SNPchips/9913_ARS1.2_777962_HD_marker_name_180910
.vcf.gz \
   -resource
GGPHDV3,known=false,training=true,truth=true,prior=15.0:SNPchips/9913_ARS1.2_139977_GGPHDV3_marker_na
me_180910.vcf.gz \
   -resource
ZLD2,known=false,training=true,truth=true,prior=15.0:SNPchips/9913_ARS1.2_18206_ZLD2_marker_name_180910.
vcf.gz \
   -resource
ZLD4,known=false,training=true,truth=true,prior=15.0:SNPchips/9913_ARS1.2_20503_ZLD4_marker_name_180910.
vcf.gz \
   -resource
GGPF250,known=false,training=true,truth=true,prior=15.0:SNPchips/9913_ARS1.2_227234_GGPF250_marker_nam
e_180910.vcf.gz \
   -resource
GGPLDV3,known=false,training=true,truth=true,prior=15.0:SNPchips/9913_ARS1.2_26504_GGPLDV3_marker_nam
e_180910.vcf.gz \
   -resource
BOVGGPV3K,known=false,training=true,truth=true,prior=15.0:SNPchips/9913_ARS1.2_2900_BOVGGPV3K_marker
_name_180910.vcf.gz \
   -resource
GGPLDV4,known=false,training=true,truth=true,prior=15.0:SNPchips/9913_ARS1.2_30105_GGPLDV4_marker_nam
e_180910.vcf.gz \
   -resource
GGPIND35,known=false,training=true,truth=true,prior=15.0:SNPchips/9913_ARS1.2_35339_GGPIND35_marker_na
me_180910.vcf.gz \
   -resource
DAIRYULDB,known=false,training=true,truth=true,prior=15.0:SNPchips/9913_ARS1.2_4227_DAIRYULDB_marker_n
ame_180910.vcf.gz \
   -resource
BOVG50V1,known=false,training=true,truth=true,prior=15.0:SNPchips/9913_ARS1.2_47843_BOVG50V1_marker_na
me_180910.vcf.gz \
   -resource
ANGGS,known=false,training=true,truth=true,prior=15.0:SNPchips/9913_ARS1.2_49541_ANGGS_marker_name_18
0910.vcf.gz \
   -resource
SNP50V3,known=false,training=true,truth=true,prior=15.0:SNPchips/9913_ARS1.2_53218_SNP50V3_marker_name
_180910.vcf.gz \
   -resource
IDBV3,known=false,training=true,truth=true,prior=15.0:SNPchips/9913_ARS1.2_53450_IDBV3_marker_name_1809
10.vcf.gz \
```

```
    -resource
BOVMD,known=false,training=true,truth=true,prior=15.0:SNPchips/9913_ARS1.2_57134_BOVMD_marker_name_18
0910.vcf.gz \
    -resource
SNP50,known=false,training=true,truth=true,prior=15.0:SNPchips/9913_ARS1.2_58336_SNP50_marker_name_180
910.vcf.gz \
    -resource
ZOETIS1,known=false,training=true,truth=true,prior=15.0:SNPchips/9913_ARS1.2_59825_ZOETIS1_marker_name_
180910.vcf.gz \
    -resource
BOS1,known=false,training=true,truth=true,prior=15.0:SNPchips/9913_ARS1.2_648875_BOS1_marker_name_1809
10.vcf.gz \
    -resource
ZMD2,known=false,training=true,truth=true,prior=15.0:SNPchips/9913_ARS1.2_68213_ZMD2_marker_name_18091
0.vcf.gz \
    -resource
BOVLDC,known=false,training=true,truth=true,prior=15.0:SNPchips/9913_ARS1.2_6909_BOVLDC_marker_name_1
80910.vcf.gz\
    -resource
IND90KH,known=false,training=true,truth=true,prior=15.0:SNPchips/9913_ARS1.2_74150_IND90KH_marker_name_
180910.vcf.gz \
    -resource
GGP90KT,known=false,training=true,truth=true,prior=15.0:SNPchips/9913_ARS1.2_76999_GGP90KT_marker_nam
e_180910.vcf.gz \
    -resource
BOVLDV2A,known=false,training=true,truth=true,prior=15.0:SNPchips/9913_ARS1.2_7931_BOVLDV2A_marker_na
me_180910.vcf.gz \
    -resource
GGPLDV1,known=false,training=true,truth=true,prior=15.0:SNPchips/9913_ARS1.2_8762_GGPLDV1_marker_name
_180910.vcf.gz \
    -resource 1000G,known=false,training=true,truth=false,prior=10.0:1000BullGenome/ARS1.2PlusY_BQSR.vcf.gz \
    -resource dbSNP,known=true,training=false,truth=false,prior=2.0:dbSNP/cattle_dbSNP.vcf.gz
    -an DP -an QD -an FS -an MQRankSum -an SOR -an ReadPosRankSum -an InbreedingCoeff \
    -mode SNP \
    -tranche 100.0 -tranche 99.9 -tranche 99.0 -tranche 90.0 \
    -O ./VREC/CattleACDr2_output.snp.recal \
    --tranches-file ./VREC/CattleACDr2_output.snp.tranches \
    --rscript-file ./VREC/CattleACDr2_output.snp.plots.R
```

The process generated the following plot describing the composition of the different tranches:

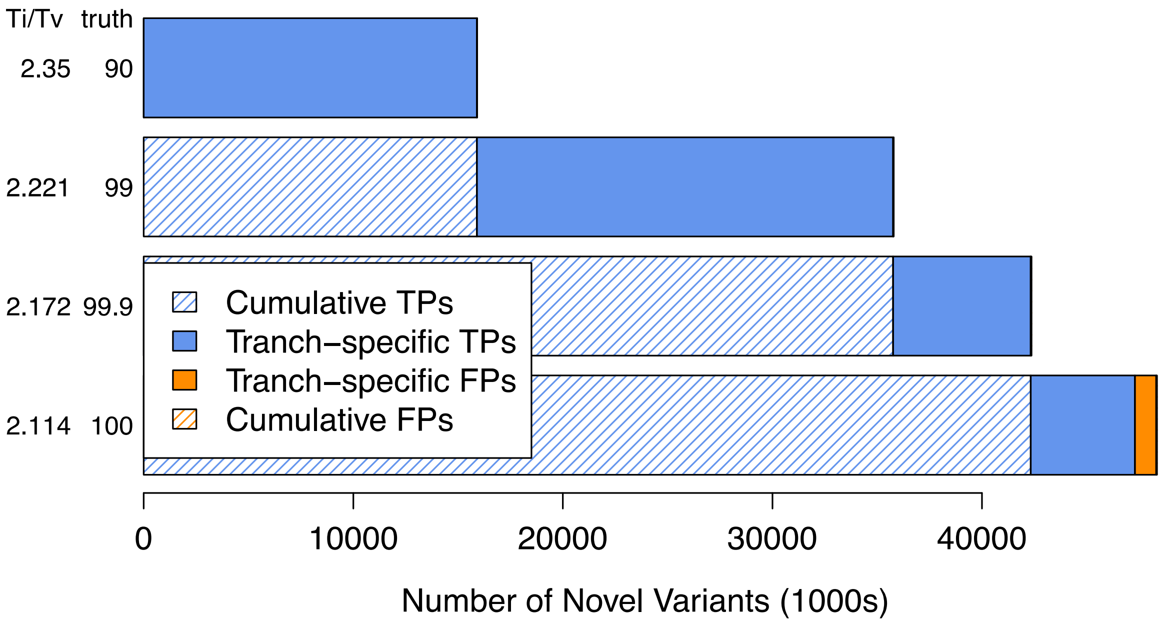

After that, we screened the plots to define the best tranche to apply for the filtering. We chose the 99 percentile, since it represented the largest chunk of variants, and with an overall high quality and low composition of false positives. The soft filter (added a flag in the FILTER field of the VCF file) has been applied using the following command:

```
gatk --java-options "-Xmx22g" ApplyVQSR \
   -R ARS-UCD1.2_Btau5.0.1Y.fasta \
   -V input.vcf.gz \
   -mode SNP \
   --recal-file ./VREC/CattleACDr2_output.snp.recal \
   --tranches-file ./VREC/CattleACDr2_output.snp.tranches \
   -O ./VREC/CattleACDr2.snprecal.vcf.gz \
   --truth-sensitivity-filter-level 99.0
```

Supplementary Table 1: Whole Genome Sequencing information of 81 water buffaloes from 7 breeds. Rows with duplicate samples are coloured using the same colour-green and yellow

Sample Name	Breed	Sequencing centre	Median Coverage
Banni_1_30x	Banni	Edinburgh Genomics (UK)	39.0X
Banni_2_30x	Banni	Edinburgh Genomics (UK)	38.0X
Banni_3_30x	Banni	Edinburgh Genomics (UK)	40.0X
Banni_4_30x	Banni	Edinburgh Genomics (UK)	38.0X
Banni_5_30x	Banni	Edinburgh Genomics (UK)	38.0X
Banni_6_30x	Banni	Edinburgh Genomics (UK)	36.0X
Bu.B.2_10x	Banni	SciGenom Labs (India)	7.0X
Bu.B.4_10x	Banni	SciGenom Labs (India)	7.0X
Bunny-0903_10x	Banni	SciGenom Labs (India)	7.0X
Bunny-0914_10x	Banni	SciGenom Labs (India)	8.0X
Bunny-0982_10x	Banni	SciGenom Labs (India)	8.0X
Bunny-960_10x	Banni	SciGenom Labs (India)	7.0X
Bhadawari_1_30x	Bhadawari	Edinburgh Genomics (UK)	40.0X
Bhadawari_2_30x	Bhadawari	Edinburgh Genomics (UK)	38.0X
Bhadawari_3_30x	Bhadawari	Edinburgh Genomics (UK)	36.0X
Bhadawari_4_30x	Bhadawari	Edinburgh Genomics (UK)	40.0X
Bhadawari_5_30x	Bhadawari	Edinburgh Genomics (UK)	39.0X
Bhadawari_6_30x	Bhadawari	Edinburgh Genomics (UK)	37.0X
Bhadawari_male_30x	Bhadawari	Edinburgh Genomics (UK)	34.0X
Bhadhwari-9-369_10x	Bhadawari	SciGenom Labs (India)	9.0X
Bhadhwari-B167_10x	Bhadawari	SciGenom Labs (India)	8.0X
Bhadhwari-B254_10x	Bhadawari	SciGenom Labs (India)	8.0X
Bhadhwari-B277_10x	Bhadawari	SciGenom Labs (India)	8.0X
Bhadhwari-B278_10x	Bhadawari	SciGenom Labs (India)	8.0X

Bhadhwari-B284_10x	Bhadawari	SciGenom Labs (India)	8.0X
Female_Grantown_buffalo_30x	Italian (Mediterranean)	Edinburgh Genomics (UK)	38.0X
Kirkcaldy_female_30x	Italian (Mediterranean)	Edinburgh Genomics (UK)	35.0X
Kirkcaldy_male_30x	Italian (Mediterranean)	Edinburgh Genomics (UK)	35.0X
Lodi_female_30x	Italian (Mediterranean)	Edinburgh Genomics (UK)	34.0X
Lodi_male_30x	Italian (Mediterranean)	Edinburgh Genomics (UK)	36.0X
Male_Grantown_buffalo_30x	Italian (Mediterranean)	Edinburgh Genomics (UK)	38.0X
Jaffarabadi_1_30x	Jaffarabadi	Edinburgh Genomics (UK)	34.0X
Jaffarabadi_2_30x	Jaffarabadi	Edinburgh Genomics (UK)	37.0X
Jaffarabadi_3_30x	Jaffarabadi	Edinburgh Genomics (UK)	36.0X
Jaffarabadi_4_30x	Jaffarabadi	Edinburgh Genomics (UK)	36.0X
Jaffarabadi_5_30x	Jaffarabadi	Edinburgh Genomics (UK)	36.0X
Jaffarabadi_6_30x	Jaffarabadi	Edinburgh Genomics (UK)	35.0X
Jaffarabadi_male_30x	Jaffarabadi	Edinburgh Genomics (UK)	38.0X
Jaffrabadi-0685_10x	Jaffarabadi	SciGenom Labs (India)	8.0X
Jaffrabadi-0845_10x	Jaffarabadi	SciGenom Labs (India)	7.0X
Jaffrabadi-2304430_10x	Jaffarabadi	SciGenom Labs (India)	8.0X
Jaffrabadi-230964_10x	Jaffarabadi	SciGenom Labs (India)	6.0X
Jaffrabadi-548_10x	Jaffarabadi	SciGenom Labs (India)	8.0X
Jaffrabadi-971_10x	Jaffarabadi	SciGenom Labs (India)	7.0X
Murrah_1_30x	Murrah	Edinburgh Genomics (UK)	36.0X
Murrah_2_30x	Murrah	Edinburgh Genomics (UK)	38.0X
Murrah_3_30x	Murrah	Edinburgh Genomics (UK)	38.0X
Murrah_4_30x	Murrah	Edinburgh Genomics (UK)	36.0X
Murrah_5_30x	Murrah	Edinburgh Genomics (UK)	39.0X
Murrah_6_30x	Murrah	Edinburgh Genomics (UK)	38.0X
Murraha-2984_10x	Murrah	SciGenom Labs (India)	8.0X
Murraha-RASM-28_10x	Murrah	SciGenom Labs (India)	8.0X
Murraha-RASM2_10x	Murrah	SciGenom Labs (India)	8.0X
Murraha-RRP277_10x	Murrah	SciGenom Labs (India)	8.0X

Murraha-RSM-36_10x	Murrah	SciGenom Labs (India)	9.0X
Murraha-RSM-3B_10x	Murrah	SciGenom Labs (India)	8.0X
Pandharpuri-62222_10x	Pandharpuri	SciGenom Labs (India)	8.0X
Pandharpuri-M240_10x	Pandharpuri	SciGenom Labs (India)	8.0X
Pandharpuri-M256_10x	Pandharpuri	SciGenom Labs (India)	11.0X
Pandharpuri-M257_10x	Pandharpuri	SciGenom Labs (India)	8.0X
Pandharpuri-M73_10x	Pandharpuri	SciGenom Labs (India)	7.0X
Pandharpuri-M7_10x	Pandharpuri	SciGenom Labs (India)	8.0X
Pandharpuri_1_30x	Pandharpuri	Edinburgh Genomics (UK)	37.0X
Pandharpuri_2_30x	Pandharpuri	Edinburgh Genomics (UK)	38.0X
Pandharpuri_3_30x	Pandharpuri	Edinburgh Genomics (UK)	40.0X
Pandharpuri_4_30x	Pandharpuri	Edinburgh Genomics (UK)	41.0X
Pandharpuri_5_30x	Pandharpuri	Edinburgh Genomics (UK)	40.0X
Pandharpuri_6_30x	Pandharpuri	Edinburgh Genomics (UK)	39.0X
Pandharpuri_female_30x	Pandharpuri	Edinburgh Genomics (UK)	28.0X
Su.B.1_10x	Surti	SciGenom Labs (India)	8.0X
Su.B.3_10x	Surti	SciGenom Labs (India)	6.0X
Surti-078_10x	Surti	SciGenom Labs (India)	8.0X
Surti-214_10x	Surti	SciGenom Labs (India)	8.0X
Surti-251_10x	Surti	SciGenom Labs (India)	8.0X
Surti-B367_10x	Surti	SciGenom Labs (India)	8.0X
Surti_1_30x	Surti	Edinburgh Genomics (UK)	35.0X
Surti_2_30x	Surti	Edinburgh Genomics (UK)	38.0X
Surti_3_30x	Surti	Edinburgh Genomics (UK)	34.0X
Surti_4_30x	Surti	Edinburgh Genomics (UK)	36.0X
Surti_5_30x	Surti	Edinburgh Genomics (UK)	34.0X
Surti_6_30x	Surti	Edinburgh Genomics (UK)	38.0X

Supplementary Table 2: Physical, milk and reproduction related traits for different water buffalo breeds present in the study

Breed	wither height males	wither height females	lactation length AVG	Milk yield per lactation (kg) AVG	milk fat AVG	birth weight males	birth weight females	age first parturition AVG	parturition interval AVG
Banni	138	137	301	2857	6	NA	NA	40	372
Bhadawari	128	124	280	900	7.5	27	25	49	475
Jafarabadi	150	140	289	2336	8.5	30	27.5	51	440
Murrah	142	133	300	1828	7.2	32.6	29.4	44	450
Pandharpuri	NA	130	330	1500	7	28	26	42	465
Surti	130	125	340	1400	7.9	26	24	56	535

Supplementary Table 3: Source data for cattle selective sweep enrichment analysis

MAPPED_TRAIT	n	Perm_mean	Perm_sd	z	z_P	z_P_fdr	metric
body height	2181	63.6982	6.237195445	3.415284993	6.37E-04	0.025486124	XP-EHH
red blood cell distribution width	493	65.4207	7.944483809	3.093882572	0.001975557	0.040655368	XP-CLR
body height	2181	256.3524	14.79469846	3.085402526	0.002032768	0.040655368	XP-CLR
waist-hip ratio	410	61.3255	7.542469001	2.741078551	0.006123787	0.081650487	XP-CLR
balding measurement	566	18.5745	3.976484753	2.621787998	0.008746982	0.174939647	XP-EHH
leukocyte count	574	24.838	4.562218472	2.446616721	0.01442041	0.192272129	XP-EHH
balding measurement	566	63.9946	7.815144176	2.303911431	0.021227616	0.212276159	XP-CLR
leukocyte count	574	81.7381	8.803366306	1.96082946	0.04989892	0.372149958	XP-CLR
mean corpuscular hemoglobin	597	22.4755	4.349907116	1.959697017	0.050031214	0.437822204	XP-EHH
triglyceride measurement	405	11.1062	3.178605341	-1.92103119	0.054727775	0.437822204	XP-EHH
vital capacity	461	66.1832	7.891062327	1.877668606	0.060426514	0.372149958	XP-CLR
chronotype measurement	473	59.2093	7.477103999	1.844390556	0.065126243	0.372149958	XP-CLR
blood protein measurement	1372	120.7796	10.42204806	1.748255227	0.080419842	0.399621113	XP-CLR
heel bone mineral density	1576	151.2564	11.64231993	1.695847573	0.08991475	0.399621113	XP-CLR
self reported educational attainment	1510	45.9834	5.630709984	1.601325592	0.109304824	0.694401754	XP-EHH
schizophrenia	597	22.6888	4.319747745	-1.548423749	0.121520307	0.694401754	XP-EHH
eosinophil count	511	71.9093	8.282169579	1.45984695	0.144332142	0.577328567	XP-CLR
erythrocyte count	637	88.7215	9.044430215	1.357575846	0.174598312	0.634902951	XP-CLR
cognitive function measurement	420	19.4434	4.10605436	1.353269955	0.175969404	0.758049928	XP-EHH
coronary artery disease	395	14.6729	3.566255376	-1.310309977	0.190090994	0.758049928	XP-EHH
body mass index	1264	44.0845	5.577191969	-1.270262892	0.203991002	0.758049928	XP-EHH
vital capacity	461	20.7781	4.16035951	1.255155952	0.209422152	0.758049928	XP-EHH
intraocular pressure measurement	338	36.0316	5.864711495	1.188191441	0.234757995	0.740676183	XP-CLR
total cholesterol measurement	417	10.3968	3.077319735	1.170889056	0.241643375	0.758049928	XP-EHH

type II diabetes mellitus	527	52.8779	7.128679639	1.13935545	0.254554929	0.740676183	XP-CLR
diastolic blood pressure	519	65.2536	7.830709996	1.116935757	0.264021794	0.740676183	XP-CLR
waist-hip ratio	410	19.6145	4.080876757	1.074646519	0.282533016	0.758049928	XP-EHH
mean corpuscular hemoglobin	597	75.3851	8.427912017	1.022186751	0.30669252	0.740676183	XP-CLR
unipolar depression	323	13.5137	3.478537384	-1.010108448	0.312443335	0.758049928	XP-EHH
type II diabetes mellitus	527	16.3155	3.760395432	0.979817167	0.327176376	0.758049928	XP-EHH
smoking status measurement	565	24.4112	4.514637611	-0.977088391	0.328525394	0.758049928	XP-EHH
mathematical ability	1134	40.6265	5.502383608	0.976576768	0.328778725	0.758049928	XP-EHH
systolic blood pressure	969	113.0451	10.19742442	0.976217091	0.328956895	0.740676183	XP-CLR
forced expiratory volume, response to bronchodilator	557	7.5025	2.623562856	0.951949748	0.341122468	0.758049928	XP-EHH
self reported educational attainment	1510	170.5939	12.29699575	-0.942823779	0.345771047	0.740676183	XP-CLR
age at menarche	350	44.0999	6.586900539	-0.926065296	0.354412048	0.740676183	XP-CLR
unipolar depression	323	43.1199	6.588191286	0.89252114	0.372113667	0.740676183	XP-CLR
high density lipoprotein cholesterol measurement	510	12.8989	3.392938254	-0.854392206	0.392887713	0.795582211	XP-EHH
neuroticism measurement	721	54.8193	7.251682991	0.852312492	0.394040675	0.740676183	XP-CLR
coronary artery disease	395	46.2945	6.695465083	0.852143941	0.394134206	0.740676183	XP-CLR
glomerular filtration rate	396	14.9363	3.623224405	0.845572799	0.397791105	0.795582211	XP-EHH
intelligence	1245	117.6451	10.45943048	-0.826536399	0.408499879	0.740676183	XP-CLR
chronotype measurement	473	17.8337	3.938425501	0.803950716	0.421425439	0.802715121	XP-EHH
body mass index	1264	154.6851	11.69851373	0.796246448	0.425888805	0.740676183	XP-CLR
eosinophil count	511	21.8458	4.278440471	0.737231246	0.460981735	0.804802926	XP-EHH
risk-taking behaviour	340	13.4538	3.467487261	0.734306951	0.462761682	0.804802926	XP-EHH
red blood cell distribution width	493	20.2854	4.150646893	0.654018535	0.513099864	0.85516644	XP-EHH
total cholesterol measurement	417	32.4078	5.610601309	0.64025223	0.522008632	0.870014386	XP-CLR
systolic blood pressure	969	32.8947	5.004229825	0.620535049	0.534905586	0.855848938	XP-EHH
FEV/FEC ratio	565	78.8365	8.759522857	0.589472747	0.555544189	0.875853302	XP-CLR
glomerular filtration rate	396	46.7822	6.68432728	-0.565831061	0.571508629	0.875853302	XP-CLR

diastolic blood pressure	519	20.3579	4.204304367	-0.560829996	0.574913436	0.876191125	XP-EHH
triglyceride measurement	405	35.1579	5.879576901	-0.53709647	0.591200979	0.875853302	XP-CLR
response to bronchodilator, FEV/FEC ratio	794	13.8645	3.473578317	-0.536766363	0.591429009	0.876191125	XP-EHH
wellbeing measurement	347	43.2725	6.421445884	0.424748577	0.671019968	0.958599954	XP-CLR
breast carcinoma	448	16.4576	3.816996095	0.404087393	0.686148455	0.952498844	XP-EHH
FEV/FEC ratio	565	24.7369	4.521849522	-0.384112738	0.700894891	0.952498844	XP-EHH
erythrocyte count	637	26.583	4.608712947	-0.343479843	0.731237502	0.952498844	XP-EHH
mathematical ability	1134	144.2799	11.33552846	0.328180553	0.742775151	0.986209018	XP-CLR
pulse pressure measurement	528	20.681	4.155955509	0.317375871	0.750958414	0.952498844	XP-EHH
adolescent idiopathic scoliosis	803	126.3936	10.71921495	-0.316590349	0.75155446	0.986209018	XP-CLR
blood protein measurement	1372	33.3984	5.142132008	-0.271949456	0.785660884	0.952498844	XP-EHH
age at menarche	350	14.0446	3.515685828	0.27175352	0.785811546	0.952498844	XP-EHH
schizophrenia	597	74.7749	8.449293997	0.263347447	0.792282793	0.986209018	XP-CLR
adolescent idiopathic scoliosis	803	39.2054	5.363234777	-0.224752421	0.822171882	0.954185894	XP-EHH
smoking status measurement	565	78.9499	8.700181567	-0.224121759	0.822662562	0.986209018	XP-CLR
pulse pressure measurement	528	65.5785	7.843251201	-0.201255826	0.840498541	0.986209018	XP-CLR
risk-taking behaviour	340	43.2366	6.442543836	-0.191942815	0.847786999	0.986209018	XP-CLR
neuroticism measurement	721	15.412	3.664941907	0.160439105	0.872535187	0.954185894	XP-EHH
wellbeing measurement	347	13.558	3.491683718	-0.159808289	0.873032094	0.954185894	XP-EHH
high density lipoprotein cholesterol measurement	510	40.9392	6.206638027	-0.151321858	0.879721827	0.986209018	XP-CLR
intraocular pressure measurement	338	11.4405	3.204010974	-0.137483924	0.890648299	0.954185894	XP-EHH
low density lipoprotein cholesterol measurement	374	9.6428	3.040416513	0.117483903	0.906476599	0.954185894	XP-EHH
breast carcinoma	448	56.4043	7.444674864	0.080016926	0.936223794	0.986209018	XP-CLR
heel bone mineral density	1576	39.6928	5.389539208	0.056999307	0.954545747	0.979021279	XP-EHH
response to bronchodilator, FEV/FEC ratio	794	44.6349	6.609717908	0.055236851	0.955949771	0.986209018	XP-CLR

low density lipoprotein cholesterol measurement	374	30.1383	5.406061011	-0.025582397	0.979590426	0.986209018	XP-CLR
intelligence	1245	31.1081	4.955912681	-0.021812329	0.982597659	0.982597659	XP-EHH
forced expiratory volume, response to bronchodilator	557	24.1023	4.898043874	-0.020885889	0.983336683	0.986209018	XP-CLR
cognitive function measurement	420	63.134	7.752254752	-0.017285294	0.986209018	0.986209018	XP-CLR

Supplementary Table 4: Data for obtaining optimal filtering criteria in cattle based on genotype quality and call rate

Genotype quality	Call rate	Number of Variants
10	50	64684639
15	50	64614210
20	50	64351590
25	50	63552861
30	50	62142509
10	75	64083453
15	75	63521900
20	75	56383202
25	75	11215339
30	75	2176263
10	80	63611896
15	80	62315516
20	80	43093015
25	80	2143038
30	80	212735
10	90	59220842
15	90	48495769
20	90	1711370
25	90	28540
30	90	25138
