# Supplementary material for: Whole genome analysis of water buffalo and global cattle breeds highlights convergent signatures of domestication
Source: Nat Commun. 2020 Sep 21;11:4739. doi: 10.1038/s41467-020-18550-1 (PMC7505982; doi:10.1038/s41467-020-18550-1)
Supplement: Supplementary file 3 — Reporting Summary [file 41467_2020_18550_MOESM3_ESM.pdf]

nature research

Corresponding author(s): James Prendergast

Last updated by author(s): 21/08/20

Reporting Summary

Nature Research wishes to improve the reproducibility of the work that we publish. This form provides structure for consistency and transparency in reporting. For further information on Nature Research policies, see our Editorial Policies and the Editorial Policy Checklist.

Statistics

For all statistical analyses, confirm that the following items are present in the figure legend, table legend, main text, or Methods section.

n/a	Confirmed	
☐	☒	The exact sample size (*n*) for each experimental group/condition, given as a discrete number and unit of measurement
☒	☐	A statement on whether measurements were taken from distinct samples or whether the same sample was measured repeatedly
☐	☒	The statistical test(s) used AND whether they are one- or two-sided<br>*Only common tests should be described solely by name; describe more complex techniques in the Methods section.*
☒	☐	A description of all covariates tested
☐	☒	A description of any assumptions or corrections, such as tests of normality and adjustment for multiple comparisons
☐	☒	A full description of the statistical parameters including central tendency (e.g. means) or other basic estimates (e.g. regression coefficient) AND variation (e.g. standard deviation) or associated estimates of uncertainty (e.g. confidence intervals)
☐	☒	For null hypothesis testing, the test statistic (e.g. *F*, *t*, *r*) with confidence intervals, effect sizes, degrees of freedom and *P* value noted<br>*Give P values as exact values whenever suitable.*
☒	☐	For Bayesian analysis, information on the choice of priors and Markov chain Monte Carlo settings
☒	☐	For hierarchical and complex designs, identification of the appropriate level for tests and full reporting of outcomes
☐	☒	Estimates of effect sizes (e.g. Cohen's *d*, Pearson's *r*), indicating how they were calculated

Our web collection on statistics for biologists contains articles on many of the points above.

Software and code

Policy information about availability of computer code

Data collection	No software was used
Data analysis	BWA 0.7.17, GATK 4.0.4.0 or 4.0.11.0, Picard 2.14.0, SAMtools 1.6 or 1.9, Python 2.7, VCFtools 0.1.13, BCFtools 1.6, TreeMix 1.13, PLINK 1.90b4 64-bit, FigTree 1.4.4, Graphlan 1.1.3 , PHYLIP 3.698, Hapbin 1.3, JBrowse 1.16, BEDtools 2.27.1, ggplot 3.3.2, SNeP 1.1, Beagle 5.0, xpclr 1.1.2, BamTools 2.4.2, VEP v95, SnpEff 4.3e or 4.3t, tabix 0.2.4

For manuscripts utilizing custom algorithms or software that are central to the research but not yet described in published literature, software must be made available to editors and reviewers. We strongly encourage code deposition in a community repository (e.g. GitHub). See the Nature Research guidelines for submitting code & software for further information.

Data

Policy information about availability of data

All manuscripts must include a data availability statement. This statement should provide the following information, where applicable:
- Accession codes, unique identifiers, or web links for publicly available datasets
- A list of figures that have associated raw data
- A description of any restrictions on data availability

All XP-EHH and XP-CLR scores used in this analysis are viewable and downloadable at our BOmA browser (https://www.bomabrowser.com/waterbuffalo/). The raw sequencing data for the novel water buffalo and cattle samples have been deposited at the European Nucleotide Archive83 (ENA) with study IDs PRJEB39591 [https://www.ebi.ac.uk/ena/browser/view/PRJEB39591], PRJEB39330 [https://www.ebi.ac.uk/ena/browser/view/PRJEB39330] and PRJEB39924 [https://www.ebi.ac.uk/ena/browser/view/PRJEB39924]. The accessions for the previously published datasets can be found in Supplementary Data 8. Additional source data for Figures 1B and 4 and Supplementary Figures 2 are provided in the Source Data and Code file.

Field-specific reporting

Please select the one below that is the best fit for your research. If you are not sure, read the appropriate sections before making your selection.

☒ Life sciences ☐ Behavioural & social sciences ☐ Ecological, evolutionary & environmental sciences

For a reference copy of the document with all sections, see nature.com/documents/nr-reporting-summary-flat.pdf

Life sciences study design

All studies must disclose on these points even when the disclosure is negative.

Sample size	79 for the Indian water buffalo, 427 for the cattle (294 after filtering). The number of water buffalo genomes sequenced was a compromise between costs and coverage of breeds and our prior experience as developers of the hapbin software of the minimum number of samples per breed required to obtain reasonable results
Data exclusions	Individuals were excluded from downstream analyses if not matching the minimum individual call rate or the maximum relatedness. Variants were excluded first by the VQSR/hard filtering, then by call rate, minor allele frequencies, Genotype quality and biallelicity.
Replication	Where doable, bootstrap of analyses have been performed to assess the stability of the analyses (i.e. IBS-based phylogeny)
Randomization	Individuals were sampled to be representative of the diversity of the respective species (Indian water buffalo breeds with their geographical location, and cattle representative of the different known hotspots of diversity), coupled by a genotype-based downstream refinement.
Blinding	All samples for each species were analysed identically and using the same pipelines irrespective of breed or sampling location.

Reporting for specific materials, systems and methods

We require information from authors about some types of materials, experimental systems and methods used in many studies. Here, indicate whether each material, system or method listed is relevant to your study. If you are not sure if a list item applies to your research, read the appropriate section before selecting a response.

Materials & experimental systems

n/a	Involved in the study	
☒	☐	Antibodies
☒	☐	Eukaryotic cell lines
☒	☐	Palaeontology and archaeology
☐	☒	Animals and other organisms
☒	☐	Human research participants
☒	☐	Clinical data
☒	☐	Dual use research of concern

Methods

n/a	Involved in the study	
☒	☐	ChIP-seq
☒	☐	Flow cytometry
☒	☐	MRI-based neuroimaging

Animals and other organisms

Policy information about studies involving animals; ARRIVE guidelines recommended for reporting animal research

Laboratory animals	The study did not involve laboratory animals
Wild animals	The study did not involve wild animals
Field-collected samples	The Indian water buffalo samples were collected from farms associated with the BAIF research institute. The Mediterranean buffalo were collected from a farm in Scotland. No other parameters were collected across samples.
Ethics oversight	All sampling was done in accordance with the regulations of the relevant local research institutes (BAIF and ILRI) and ethics approval was obtained from The Roslin Institute's and the University of Edinburgh's Protocols and Ethics Committees. All animal work was carried out in accordance with the regulations of the Animals (Scientific Procedures) Act 1986.

Note that full information on the approval of the study protocol must also be provided in the manuscript.
