# Supplementary material for: Whole genome analysis of water buffalo and global cattle breeds highlights convergent signatures of domestication
Source: Nat Commun. 2020 Sep 21;11:4739. doi: 10.1038/s41467-020-18550-1 (PMC7505982; doi:10.1038/s41467-020-18550-1)
Supplement: Supplementary file 4 — Description of Additional Supplementary Files [file 41467_2020_18550_MOESM4_ESM.pdf]

Description of Additional Supplementary Files

File name: Supplementary data 1

Description: The location of water buffalo XP-EHH peaks

File name: Supplementary data 2

Description: The location of water buffalo XP-CLR peaks

File name: Supplementary data 3

Description: The overlap between water buffalo XP-EHH and XP-CLR peaks across peak calling thresholds

File name: Supplementary data 4

Description: The associations between water buffalo phenotypes and XP-EHH scores at different loci

File name: Supplementary data 5

Description: The location of cattle XP-EHH peaks

File name: Supplementary data 6

Description: The location of cattle XP-CLR peaks

File name: Supplementary data 7

Description: The overlap of metrics across species and thresholds

File name: Supplementary data 8

Description: The publically available cattle datasets used in this study

File name: Supplementary data 9

Description: The metrics of the cattle samples

File name: Supplementary Software 1

Description: Code for performing downstream analyses
