# Supplementary material for: Whole genome analysis of water buffalo and global cattle breeds highlights convergent signatures of domestication
Source: Nat Commun. 2020 Sep 21;11:4739. doi: 10.1038/s41467-020-18550-1 (PMC7505982; doi:10.1038/s41467-020-18550-1)
Supplement: Supplementary file 14 — Supplementary Software 1 [file 41467_2020_18550_MOESM14_ESM.zip › Code/WaterBuffalo/Variant_Calling_and_filtering_commands.docx]

| **Job** | **Tools with version** | **Parameter** |
| --- | --- | --- |
| **Alignment** | BWA-MEM 0.7.17, SAMtools view 1.6 (for conversion from SAM to BAM) | Defaults for bwa index (indexing the reference genome)  Defaults for bwa mem (alignment)  –b (for SAMtools view) |
| **BAM file sorting** | Samtools sort 1.6 | Defaults |
| **Marking Duplicates** | Picard 2.14.0 MarkDuplicates | ASSUME_SORTED=true VALIDATION_STRINGENCY=SILENT MAX_FILE_HANDLES_FOR_READ_ENDS_MAP=1024 TMP_DIR=<temp_directory> METRICS_FILE=<metrics_filename> |
| **Adding read groups** | Picard 2.14.0 AddOrReplaceReadGroups | RGLB=library RGPL=illumina RGPU=barcode RGSM=<sample_name> CREATE_INDEX=true |
| **Call variants per sample in GVCF mode** | GATK 4.0.4.0 Haplotypecaller | -ERC GVCF |
| **Consolidate GVCFs** | GATK 4.0.4.0 GenomicsDBImport | --TMP_DIR <temp_directory> --sample-name-map <sample_name_mapping_file> --reader-threads 2 --genomicsdb-workspace-path <chromosomes_and unplaced_scaffolds_name> -L <chromosomes_and unplaced_scaffolds_name> |
| **Joint call cohort** | GATK 4.0.4.0 GenotypeGVCF | -new-qual -V gendb:// <chromosomes_and unplaced_scaffolds_name> |
| **Concatenate variants called per chromosome/unplaced_scaffold into a final multi-sample VCF file** | Picard 2.14.0 GatherVcfs | Defaults |
| **Index the final VCF file** | Tabix | Defaults |
| **Get only biallelic SNVs** | BCFtools view 1.6 | -v snps -m2 -M2 |
| **Hard filter biallelic variants** | BCFtools filter 1.6 | -i 'QD >= 15 & FS <= 60 & SOR <= 2 & MQ >= 50 & MQRankSum >= -2.5 & ReadPosRankSum >= -2.5' |
