# Supplementary material for: Whole genome analysis of water buffalo and global cattle breeds highlights convergent signatures of domestication
Source: Nat Commun. 2020 Sep 21;11:4739. doi: 10.1038/s41467-020-18550-1 (PMC7505982; doi:10.1038/s41467-020-18550-1)
Supplement: Supplementary file 2 — Peer Review File [file 41467_2020_18550_MOESM2_ESM.pdf]

Reviewers' comments:

Reviewer #1 (Remarks to the Author):

The authors identified signatures of selection in whole genome sequence data of 6 Indian water buffalo breeds and compared them to those found in previous studies as well as in 13 global cattle breeds. They highlight signatures pointing towards convergent evolution first with a handful where there is overlap with ones found in other domesticated or model organisms including humans and then compare to their results in global cattle. The manuscript is very well written and technically sound. I particularly appreciate the detailed methods section with software versioned, cited, and parameters given. My only major criticism is the reliance upon a single method to detect signatures. The introduction mentions "more recent selective sweeps between domesticated breeds…" yet the chosen test, XP-EHH performs best if the selected allele is close to or at fixation. Different tests are better suited to the detection of more recent selection. A more minor criticism is that it would be nice to see some more complete reporting of results in a table for all regions specifically discussed in the text and supplementary tables for the rest.

Specific comments:

Line 53: Consider rephrasing: "phenotypic and production phenotypes", "morphological and production phenotypes" maybe?

Line 108: I know the assembly is named in the Methods but it would be nice to have it named here where first listed, particularly since more high-quality chromosome level assemblies are being released for water buffalo. https://www.biorxiv.org/content/10.1101/618785v2

Line 117: Not necessary, but would be nice if the color coding of supplementary figure 2 matched figure 1.

Lines 154-160: Would like to see XP-EHH scores in supplementary table or at least see the top 15 mentioned.

Lines 239-241: "The breed with the lowest average birth weight, Surti, displayed the greatest levels of haplotype homozygosity at the GHR locus, with a general trend of a reduction in haplotype homozygosity across the lower birth weight breeds" The first and second parts of this sentence disagree, there is an increase in homozygosity in the lower birth weight breeds right?

Line 347: "Four of the fifteen cattle regions had at least one breed comparison with a significantly elevated median XP-EHH" only two mentioned in the text and three in the figure.

Line 351: Having coordinates in the text would make it easier to match text with sup figure 6.

Line 431: Need details regarding sequencing including what type of sequencer and what type of sequencing libraries were used.

Line 503-504: Data from FAO was used, was this data not available for the sequenced individuals?
Line 515: Include sequencing details (sequencer, coverage) for animals from SRA (when available) in supplemental table. Was there a coverage threshold used for the inclusion of the 427 cattle?

Line 518: Need sequencing details for these as well.

Reviewer #2 (Remarks to the Author):

This manuscript explores selection sweeps between different Water Buffalo breeds as well as across bovid and links it to phenotypic traits. Also, the regions are linked to human GWAS results, making it a comprehensive across species comparison.
Although the regions found are known to have impact on for instance stature phenotypes across mammals, they show that the selection in these regions occurred independently and resulted in the same mutation, which is a rather novel finding in my humble experience.

In general, I find it a well written paper and am pleased with the extensive work that has been done. It has relevance and importance in the field.

My only criticism would be that the material & methods section could be better linked to the results section. Most, but not all descriptions lead to the information provided in the results section. Most technical details are provided but it is not always clear why and for which results that particular part was used. Some are indicated below in my line by line comments.

L107 Please add reference for the genome assembly that showed the actual high quality
L198 I like to suggest to write TSS in full as not all readers may be aware of it
L430 Alignment is also included in this section
L431 Better not start a sentence with a number or write it in full
L431-432 What type of sequence data was it? E.g. sequencing platform, paired end, read length?
L446 I suggest to change 'obtained' into 'retained for further analyses'
L452 Please provide the definition of Ti/Tv ratio. In human it should be 2-2.1, is there an expectation for water buffalo (or cattle)? Is it a good thing it increased?
L380-382 Not in human?
L487 Beagle 5.0 was used with default settings. Default settings have a Ne of 1,000,000 because it is designed for outbred humans populations. It has been shown that imputation in livestock and crops is severely impacted by this default leading to imputation errors, see Pook et al. 2020 (G3).
What is your expectation of Ne for water buffalo?
L517 Why add the Y chromosome if you are only interested in autosomes?
L528 Please define VQSR
L536-540 Were indels used in the analyses? Also there were 64M variants after filtering, while in line 277 only 11M were used.

Reviewer #3 (Remarks to the Author):

In the manuscript "Whole genome analysis of water buffalo and global cattle breeds highlights convergent signature of recent selection across domesticated species" The authors describe a homozygosity based selection scan looking for regions that have been important in breed formation in buffalo and cattle. Upon reading the title, I was hoping that the authors had looked more broadly at the regions under selection for domestication in the two species rather than those that are preferred for breed formation. Not surprisingly they identified loci with possible association with size and coat since these are the first two traits one sees when identifying a breed in any species likely because these are qualities easily distinguished by humans wishing to subset a group of similar animals.
It was interesting to see that an identical mutation in the ASIP gene was identified in the buffalo that has been found to alter coat color in dogs. I'm not sure that convergent evolution is the correct term for this since this is a mutation that is likely selected by humans in both cases and not an aspect of evolution of the species. This is more indicative of a tolerated polymorphic site in this gene. One major concern regarding this discussion point is the lack of any phenotypic data to go along with what is

expected to be a mutation that would change coat color. Based on the other species data, the buffalo with this mutation should be solid black but the pictures and the descriptions give no indication of this. Also, the ASIP gene appears to be on the far boundary of the region showing strong selection. What are the other genes here and why aren't they discussed?
This is the same for the GHR region, this gene is on one edge of the locus, the selection looks stronger in the neighboring region and the other genes are not discussed. I think it would help to have a table of the regions under selection between buffalo breeds and all the genes that could be targets rather than just the one favorite gene. Also more description of the breeds and how and why they differ is required.
I do not see anywhere if there was a minimum window size considered for a region under selection and what the average size of these regions was.
Admixture analysis was done for buffalo and cattle breeds but the results are never mentioned in the manuscript. These figures could be removed since they are not contributing to the story. If they are retained, they should be discussed and the labeling needs to be improved. It is very difficult to see where the boundaries for the breeds are.
In the correlation between selection for a locus around GHR and size, the result is given for birth weight but not for height. In the figure, height looks more significant but not numbers are given. This should be acknowledged and discussed.
In figure 6, two of the regions are mentioned in the manuscript but the third is not. What is this region, why is it included but not mentioned?
The discussion ends with talk of differences in coat type but there is no phenotypic data to say if the buffalo breeds have differences. This needs to be added and if there is none, then provide examples of why this should be important.
What was considered significant in the overlap between regions of selection in buffalo/cattle and human GWAS? GWAS loci are often quite large and the database reports multiple SNPs from the same study. In addition, many studies never lead to actual mutations. Was this considered in your analysis and how was it accounted for? Can you provide the average size of the GWAS loci that you overlapped and how many variants on average were in the overlap?
Other notes:
Line 68, remove though from the sentence "there have though been few…"
Line 94 remove "down to" in the sentence "..including down to individual functional…"
Also, should these be potentially functional?
The Supplementary tables need to be reformatted to be able to read across the columns. The columns are unnecessarily wide and can be narrowed to make them fit on one page.
Lines 132-133 you write "these breeds are morphologically very similar to each other with similar production potential." Based on the limited data you have provided about the breeds these don't look any more similar than many other pairs. Can you give more description here?
Line 232 – remove "the difference in" from "strongly correlated with the difference in mean birth weight differences between…" or, alternately, remove "differences".
Figure 4. I can't really see the difference between hexagon and pentagon on this figure. Can you use a more discernable shape?
Line 503: you mention fat content here but this is not provided in figure S1 with the other traits. You do not mention parturition interval which is included in the figure.
Line 545: talks about Admixture analysis done in cattle breeds but this data is not shown or discussed. It seems that all references to admixture analysis can be removed from this manuscript.

RESPONSE TO REVIEWERS

Reviewer #1 (Remarks to the Author):

The authors identified signatures of selection in whole genome sequence data of 6 Indian water buffalo breeds and compared them to those found in previous studies as well as in 13 global cattle breeds. They highlight signatures pointing towards convergent evolution first with a handful where there is overlap with ones found in other domesticated or model organisms including humans and then compare to their results in global cattle. The manuscript is very well written and technically sound. I particularly appreciate the detailed methods section with software versioned, cited, and parameters given. My only major criticism is the reliance upon a single method to detect signatures. The introduction mentions "more recent selective sweeps between domesticated breeds…" yet the chosen test, XP-EHH performs best if the selected allele is close to or at fixation. Different tests are better suited to the detection of more recent selection.

Many thanks for your comment. We have now updated the analysis throughout to also include XP-CLR results. These complementary statistics have been widely used to successfully compare livestock populations (e.g. see https://bmcgenet.biomedcentral.com/articles/10.1186/s12863-016-0430-1). We found that the majority of the loci detected using XP-EHH were also detected by XP-CLR in both water buffalo and cattle (see lines 157-161 and 295-299 for more details).

A more minor criticism is that it would be nice to see some more complete reporting of results in a table for all regions specifically discussed in the text and supplementary tables for the rest.

We have now updated the text and included new supplementary tables (Supplementary Tables 2, 3, 7 and 8) listing all peaks for both statistics and both species as well as the genes they contain.

Specific comments:

Line 53: Consider rephrasing: "phenotypic and production phenotypes", "morphological and production phenotypes" maybe?

Thanks for the suggestion, we have changed this accordingly (at line 52).

Line 108: I know the assembly is named in the Methods but it would be nice to have it named here where first listed, particularly since more high-quality chromosome level assemblies are being released for water buffalo.https://www.biorxiv.org/content/10.1101/618785v2

We have updated this and included the relevant references at lines 82 and 106.

Line 117: Not necessary, but would be nice if the color coding of supplementary figure 2 matched figure 1.

Thanks for the suggestion. This has been updated (see Supplementary Figure 2).

Lines 154-160: Would like to see XP-EHH scores in supplementary table or at least see the top 15 mentioned.

Have now added these as Supplementary Table 2-3 and 7-8. Also we have uploaded all XP-EHH and XP-CLR scores for both species to our BOMA browser so that they can be viewed or downloaded for all breed comparisons in a particular region.

Lines 239-241: "The breed with the lowest average birth weight, Surti, displayed the greatest levels of haplotype homozygosity at the GHR locus, with a general trend of a reduction in haplotype homozygosity across the lower birth weight breeds" The first and second parts of this sentence disagree, there is an increase in homozygosity in the lower birth weight breeds right?

Many thanks for spotting this, it was indeed written the wrong way round. Apologies. However, we have now updated this section (see below) and removed this sentence.

Line 347: "Four of the fifteen cattle regions had at least one breed comparison with a significantly elevated median XP-EHH" only two mentioned in the text and three in the figure.

We have now updated the text to include the location of overlapping regions and the locations of all peaks and the genes they contain are now provided as Supplementary Tables 2-3 and 7-8.

Line 351: Having coordinates in the text would make it easier to match text with sup figure 6.

Many thanks. Have now added the coordinates at lines 375-401.

Line 431: Need details regarding sequencing including what type of sequencer and what type of sequencing libraries were used.

We have now clarified these details at lines 462-468.

Line 503-504: Data from FAO was used, was this data not available for the sequenced individuals?

No unfortunately not, and we agree this would have been useful. However, as selective sweeps represent the strength of selection across the wider population and the set of ancestors of an animal, not the particular animal itself, we believe there are some merits to using population, rather than individual, level data in this type of analysis. The phenotypes measured across a large number of animals for a breed may better represent the breed and its ancestors than the smaller number of animals that were sequenced, which may for example have an unusual environmental exposure.

Line 515: Include sequencing details (sequencer, coverage) for animals from SRA (when available) in supplemental table. Was there a coverage threshold used for the inclusion of the 427 cattle?

No explicit coverage filter was applied to these samples, however, samples with very low coverage got dropped while applying the filter for individual genotype missingness. This led to all samples with a coverage less than ~5x being dropped. We have now added a spreadsheet in supplementary table 12 with the VCF-based average depth per individual.

Line 518: Need sequencing details for these as well.

Supplementary table 11 now includes details on sequencing platform for the samples.

Reviewer #2 (Remarks to the Author):

This manuscript explores selection sweeps between different Water Buffalo breeds as well as across bovid and links it to phenotypic traits. Also, the regions are linked to human GWAS results, making it a comprehensive across species comparison. Although the regions found are known to have impact on for instance stature phenotypes across mammals, they show that the selection in these regions occurred independently and resulted in the same mutation, which is a rather novel finding in my humble experience.

In general, I find it a well written paper and am pleased with the extensive work that has been done. It has relevance and importance in the field.

My only criticism would be that the material & methods section could be better linked to the results section. Most, but not all descriptions lead to the information provided in the results section. Most technical details are provided but it is not always clear why and for which results that particular part was used. Some are indicated below in my line by line comments.

Thanks. We have tried to make the methods section clearer.

L107 Please add reference for the genome assembly that showed the actual high quality

We have now added this at lines 82 and 106.

L198 I like to suggest to write TSS in full as not all readers may be aware of it

Thanks. We have now expanded this at line 201.

L430 Alignment is also included in this section

We have updated this section heading to "Water buffalo read alignment, variant calling and filtering" (line 461).

L431 Better not start a sentence with a number or write it in full

Now written in full (line 468).

L431-432 What type of sequence data was it? E.g. sequencing platform, paired end, read length?

This information has now been included at lines 462-468.

L446 I suggest to change 'obtained' into 'retained for further analyses'

Thanks. Have updated at line 484.

L452 Please provide the definition of Ti/Tv ratio. In human it should be 2-2.1, is there an expectation for water buffalo (or cattle)? Is it a good thing it increased?

We have expanded this section to cover these points at lines 491-497.

L380-382 Not in human?

Although mutations at the *GHR* and *HMGA2* genes have both been associated with important human growth phenotypes (e.g. see https://www.nature.com/articles/jhg201529, https://www.ncbi.nlm.nih.gov/pmc/articles/PMC5983672/) we could not find much evidence in the literature for these loci showing unusually strong levels of positive selection in humans. It's possible this reflects the fact that domesticated species have likely been under much stronger comparatively recent selection for body size than human populations.

L487 Beagle 5.0 was used with default settings. Default settings have a Ne of 1,000,000 because it is designed for outbred humans populations. It has been shown that imputation in livestock and crops is severely impacted by this default leading to imputation errors, see Pook et al. 2020 (G3). What is your expectation of Ne for water buffalo?

Many thanks for this. We used SNeP to estimate the Ne among the water buffalo which provided a value of 358 for the most recent generation (see lines 526-530). We therefore updated all results after setting the Ne parameter in Beagle to this value. Although it does not dramatically affect the results obtained some peaks do change in height. Many thanks for highlighting this.

L517 Why add the Y chromosome if you are only interested in autosomes?

We hope to use these variant calls in other projects and is why had included this Y chromosome even though, as the reviewer highlights, we only examined the autosomes in this analysis.

L528 Please define VQSR

We have now defined VQSR (line 594-601) and added further details on the VQSR process in Supplementary Document 1.

L536-540 Were indels used in the analyses? Also there were 64M variants after filtering, while in line 277 only 11M were used.
We have now added further details addressing these points at lines 610-616, 622 and 627.

Reviewer #3 (Remarks to the Author):

In the manuscript "Whole genome analysis of water buffalo and global cattle breeds highlights convergent signature of recent selection across domesticated species" The authors describe a homozygosity based selection scan looking for regions that have been important in breed formation in buffalo and cattle. Upon reading the title, I was hoping that the authors had looked more broadly at the regions under selection for domestication in the two species rather than those that are preferred for breed formation. Not surprisingly they identified loci with possible association with size and coat since these are the first two traits one sees when identifying a breed in any species likely because these are qualities easily distinguished by humans wishing to subset a group of similar animals.
It was interesting to see that an identical mutation in the ASIP gene was identified in the buffalo that has been found to alter coat color in dogs. I'm not sure that convergent evolution is the correct term for this since this is a mutation that is likely selected by humans in both cases

and not an aspect of evolution of the species. This is more indicative of a tolerated polymorphic site in this gene.

Thanks for the suggestion. We have renamed this convergent domestication, in that both species have likely been under selection for the same phenotypes. We don't think this is likely just a tolerated polymorphic site as the site is actually conserved across mammalian reference genomes (see new Supplementary Figure 3). So despite the conservation at this site, the exact same amino acid change linked to coat colour in dogs appears to have occurred and risen to a high frequency in breeds of both species independently.

One major concern regarding this discussion point is the lack of any phenotypic data to go along with what is expected to be a mutation that would change coat color. Based on the other species data, the buffalo with this mutation should be solid black but the pictures and the descriptions give no indication of this. Also, the ASIP gene appears to be on the far boundary of the region showing strong selection. What are the other genes here and why aren't they discussed?

Apologies, the chosen photo may be misleading regarding this. This variant is not fixed, just more common in Murrah so we wouldn't expect all animals of this breed to necessarily be black even if this polymorphism explained all the variation in coat colour between animals. Consistent with this black and non-black Murrah are observed. However, we have now swapped the image in Supplementary Figure 1 to a black Murrah to reduce this confusion.
The reviewer is right that although the ASIP polymorphism sits within the wider peak it is not directly at the centre of it. However, as the boundaries of selective sweeps are determined by factors such as the location of recombination hotspots the gene underlying any selective sweep may not necessarily sit directly at the mid-point. As shown in the updated version of Figure 2A the non-synonymous polymorphism does though sit in the comparatively narrow maximal region of the wider peak showing the highest XP-EHH scores. Given the known role of *ASIP* and the previous studies identifying it as under positive selection in other species (e.g. see https://gsejournal.biomedcentral.com/articles/10.1186/s12711-019-0512-4**) we think it is the strongest candidate in this region but we have now provided a list of the other genes in this region in Supplementary Table 2.**

This is the same for the GHR region, this gene is on one edge of the locus, the selection looks stronger in the neighboring region and the other genes are not discussed. I think it would help to have a table of the regions under selection between buffalo breeds and all the genes that could be targets rather than just the one favorite gene. Also more description of the breeds and how and why they differ is required.

We have now provided supplementary tables listing all regions and the genes falling within them (see Supplementary Table 2, 3, 7 and 8). Likewise we have added Supplementary Table 6 with more information on the traits across the breeds.

I do not see anywhere if there was a minimum window size considered for a region under selection and what the average size of these regions was.

We didn't set an explicit window size in initial peak discovery but as we have smoothed both the XP-EHH and XP-CLR scores (see updated methods at lines 539-541 and 550-560) peaks resulting from short outliers are removed. We have included peak size information in Supplementary Tables 2, 3, 7 and 8.

Admixture analysis was done for buffalo and cattle breeds but the results are never mentioned

in the manuscript. These figures could be removed since they are not contributing to the story. If they are retained, they should be discussed and the labeling needs to be improved. It is very difficult to see where the boundaries for the breeds are.

We have now removed what was Figure 1B.

In the correlation between selection for a locus around GHR and size, the result is given for birth weight but not for height. In the figure, height looks more significant but not numbers are given. This should be acknowledged and discussed.

The correlations and P values were within each panel, including for height, but this wasn't made very clear. We have now also included Supplementary Table 5 which includes all correlations and P values. To make this section more robust we extended it beyond just *GHR* to all loci and also a diverse range of traits (see lines 227-267). Another locus, around the *SQLE* gene, was more strongly associated with milk fat percentage than the *GHR* locus was with birth weight (now ranked 13th). The *SQLE* locus was also detected in the new XP-CLR analysis and we have now updated this section to focus on this locus. This has the additional advantage of helping address the reviewer's point above about expanding the focus beyond regions linked to size and coat colour.

In figure 6, two of the regions are mentioned in the manuscript but the third is not. What is this region, why is it included but not mentioned?

We have updated this section to discuss all regions (see lines 374-401).

The discussion ends with talk of differences in coat type but there is no phenotypic data to say if the buffalo breeds have differences. This needs to be added and if there is none, then provide examples of why this should be important.

We have now removed this part of the discussion.

What was considered significant in the overlap between regions of selection in buffalo/cattle and human GWAS? GWAS loci are often quite large and the database reports multiple SNPs from the same study. In addition, many studies never lead to actual mutations. Was this considered in your analysis and how was it accounted for? Can you provide the average size of the GWAS loci that you overlapped and how many variants on average were in the overlap?

In this analysis we used the locations of single nucleotide polymorphisms in the EBI GWAS catalogue lifted over to the cattle genome (https://www.ebi.ac.uk/gwas/). So each GWAS region is in effect a single variant of length 1bp, however the reviewer is right that multiple variants in a region can be associated with the same trait. Our approach though accounts for this. For each trait tested we first subsetted out the associated variants. We then counted how many of the putative selective sweep peaks overlapped one or more of these variants. If a region overlapped multiple variants linked to the same trait it was only counted once. To test whether this number of overlapping peaks was more or less than we would expect by chance given the distribution of GWAS variants in the genome we then randomly sampled the same number of regions, of the same length, from the genome and repeated the process 1000 times. This therefore accounts for the distribution of GWAS variants in the genome, as this remains constant in the permutations. To test for any enrichment, the real number of peak regions overlapping a GWAS variant was finally compared to the mean number among the permutations by calculating a Z-score. So the final P value is related to the

probability of having sampled this many peaks overlapping GWAS variants by chance, importantly given the distribution of GWAS variants in the genome and the total number and length of peaks. We have tried to make this point clearer at lines 643-645.

Other notes:
Line 68, remove though from the sentence "there have though been few…"

Thanks. That sentence has now been removed from the updated text.

Line 94 remove "down to" in the sentence "..including down to individual functional…"

Done (line 93).

Also, should these be potentially functional?

Changed to "candidate functional variants" (line 92).

The Supplementary tables need to be reformatted to be able to read across the columns. The columns are unnecessarily wide and can be narrowed to make them fit on one page.

We have now adjusted the column widths of all Supplementary Tables.

Lines 132-133 you write "these breeds are morphologically very similar to each other with similar production potential." Based on the limited data you have provided about the breeds these don't look any more similar than many other pairs. Can you give more description here?

We have now removed this sentence and provided Supplementary Table 6 with average trait measures from the FAO to enable the more precise comparison of all breeds.

Line 232 – remove "the difference in" from "strongly correlated with the difference in mean birth weight differences between…" or, alternately, remove "differences".

Many thanks. We have extensively rewritten the whole section (see section "Selective sweeps linked to production phenotypes").

Figure 4. I can't really see the difference between hexagon and pentagon on this figure. Can you use a more discernable shape?

We have now updated Figure 4 using stars instead of the pentagons.

Line 503: you mention fat content here but this is not provided in figure S1 with the other traits. You do not mention parturition interval which is included in the figure.

Please see new Supplementary Table 6.

Line 545: talks about Admixture analysis done in cattle breeds but this data is not shown or discussed. It seems that all references to admixture analysis can be removed from this manuscript.

We have now removed the admixture analysis (see above).

REVIEWERS' COMMENTS:

Reviewer #1 (Remarks to the Author):

I thank the authors for addressing my previous concerns with their manuscript. I only have a few minor suggestions.

Line 155 - Reword sentence so that it doesn't start with a number.

Lines 234-237 - Consider splitting long sentence, maybe this:
This locus mapped to a 391Kb region on chromosome 15 and was significantly associated with milk fat percentage (Figure 3A). It was also detected in the XP-CLR analysis (Figure 3B) and contains five genes: MTSS1, LOC112579209, ZNF572, SQLE and WASHC5.

Line 377 - Should be Figure 7.

Line 421 - Don't need to reference supplemental tables here.

Sup Tables 2&3 - Might be more clear to use "chromosome" instead of "seqnames" as column 1 label.

Reviewer #2 (Remarks to the Author):

I like to thank the authors for their thorough revisions. All my comments have been addressed in the revised version. I only have a few textual comments, see line by line comments below.

L155: Sentence starts with '118', please avoid starting sentences with numbers
L202: KITLG in italic, and I guess the KIT in brackets can be removed.
L570: remove 'where'
L574: please write ecdf in full

Reviewer #3 (Remarks to the Author):

The authors did a very good job of responding to and making adjustments based on the comments given. The added data tables and descriptions are very helpful. I don't have any further suggestions.

RESPONSE TO REVIEWERS

Reviewers comments

Reviewer #1 (Remarks to the Author):

I thank the authors for addressing my previous concerns with their manuscript. I only have a few minor suggestions.

Line 155 - Reword sentence so that it doesn't start with a number.

We have now reworded this sentence to "In total there were 118 and 924 loci that showed elevated XP-EHH and XP-CLR scores respectively in one or more breed comparison." at line 153.

Lines 234-237 - Consider splitting long sentence, maybe this:
This locus mapped to a 391Kb region on chromosome 15 and was significantly associated with milk fat percentage (Figure 3A). It was also detected in the XP-CLR analysis (Figure 3B) and contains five genes: MTSS1, LOC112579209, ZNF572, SQLE and WASHC5.

Have made the change as suggested at line 216.

Line 377 - Should be Figure 7.

Changed at line 303.

Line 421 - Don't need to reference supplemental tables here.

Removed reference to the supplementary files at line 336.

Sup Tables 2&3 - Might be more clear to use "chromosome" instead of "seqnames" as column 1 label.

We have replaced "seqnames" with "chromosome" in all applicable supplementary datasets/tables.

Reviewer #2 (Remarks to the Author):

I like to thank the authors for their thorough revisions. All my comments have been addressed in the revised version. I only have a few textual comments, see line by line comments below.

L155: Sentence starts with '118', please avoid starting sentences with numbers

Changed (see above).

L202: KITLG in italic, and I guess the KIT in brackets can be removed.

We believe that it is right that this isn't in italics as are referring to the protein, not the gene i.e. are talking about the protein's, not gene's, receptor. Also we believe specifying the receptor we mean in brackets may be useful for readers, though can remove if necessary.

L570: remove 'where'

We believe we can’t delete the “where” but have inserted a comma prior to it to aid reading (line 486).

L574: please write ecdf in full

Have added what ecdf stands for in brackets (line 490).

Reviewer #3 (Remarks to the Author):

The authors did a very good job of responding to and making adjustments based on the comments given. The added data tables and descriptions are very helpful. I don't have any further suggestions.
